# Explainable Machine Learning for Early Assessment of COVID-19 Risk Prediction in Emergency Departments

**DOI:** 10.1109/ACCESS.2020.3034032

**Published:** 2020-10-26

**Authors:** Elena Casiraghi, Dario Malchiodi, Gabriella Trucco, Marco Frasca, Luca Cappelletti, Tommaso Fontana, Alessandro Andrea Esposito, Emanuele Avola, Alessandro Jachetti, Justin Reese, Alessandro Rizzi, Peter N. Robinson, Giorgio Valentini

**Affiliations:** 1 Department of Computer Science “Giovanni degli Antoni,”Università degli Studi di Milano 9304 20133 Milan Italy; 2 CINI National Laboratory of Artificial Intelligence and Intelligent Systems (AIIS)Università di Roma 00185 Roma Italy; 3 Data Science Research CenterUniversità degli Studi di Milano 9304 20133 Milan Italy; 4 Dipartimento di ElettronicaInformazione e BioingegneriaPolitecnico di Milano 18981 20133 Milan Italy; 5 Radiology DepartmentFondazione IRCCS Ca Granda Ospedale Maggiore Policlinico 20122 Milan Italy; 6 Postgraduate School in RadiodiagnosticsUniversità degli Studi di Milano 9304 20122 Milan Italy; 7 Accident and Emergency DepartmentFondazione IRCCS Ca Granda Ospedale Maggiore Policlinico 20122 Milan Italy; 8 Division of Environmental Genomics and Systems BiologyLawrence Berkeley National Laboratory 1666 Berkeley CA 94720 USA; 9 The Jackson Laboratory for Genomic Medicine Farmington CT 06032 USA

**Keywords:** Associative tree, Boruta feature selection, clinical data analysis, COVID-19, generalized linear models, missing data imputation, random forest classifier, risk prediction

## Abstract

Between January and October of 2020, the severe acute respiratory syndrome coronavirus 2 (SARS-CoV-2) virus has infected more than 34 million persons in a worldwide pandemic leading to over one million deaths worldwide (data from the Johns Hopkins University). Since the virus begun to spread, emergency departments were busy with COVID-19 patients for whom a quick decision regarding in- or outpatient care was required. The virus can cause characteristic abnormalities in chest radiographs (CXR), but, due to the low sensitivity of CXR, additional variables and criteria are needed to accurately predict risk. Here, we describe a computerized system primarily aimed at extracting the most relevant radiological, clinical, and laboratory variables for improving patient risk prediction, and secondarily at presenting an explainable machine learning system, which may provide simple decision criteria to be used by clinicians as a support for assessing patient risk. To achieve robust and reliable variable selection, Boruta and Random Forest (RF) are combined in a 10-fold cross-validation scheme to produce a variable importance estimate not biased by the presence of surrogates. The most important variables are then selected to train a RF classifier, whose rules may be extracted, simplified, and pruned to finally build an associative tree, particularly appealing for its simplicity. Results show that the radiological score automatically computed through a neural network is highly correlated with the score computed by radiologists, and that laboratory variables, together with the number of comorbidities, aid risk prediction. The prediction performance of our approach was compared to that that of generalized linear models and shown to be effective and robust. The proposed machine learning-based computational system can be easily deployed and used in emergency departments for rapid and accurate risk prediction in COVID-19 patients.

## I. Introduction

Coronavirus disease 2019 (COVID-19), caused by the novel severe acute respiratory syndrome coronavirus 2 (SARS-CoV-2), emerged in Wuhan, China, in December 2019. COVID-19 quickly became a pandemic [1] and is still threatening the lives of populations worldwide. Given the promising results achieved by studies exploiting Artificial Intelligence (AI) and/or probabilistic models for outcome prediction [2]–[4] in bio-medical problems where human skill and know-how are not able to provide precise and reproducible solutions, in this worldwide health crisis, a great deal of research effort has been devoted to the development of Robotics and Data Analytics techniques [5], [6] exploiting the potentials of AI methods to either predict, monitor, and combat the virus by simulating the virus spread or the time needed to recover [7] ensuring and promoting social distancing [5], [8], identifying early COVID-19 infections, or predicting patient outcome to improve patient care [8]–[11]. Thanks to such AI techniques, wearable devices and Web applications may be used by affected individuals for self-monitoring COVID-19 related symptoms, while clinicians are aided in the diagnosis of COVID-19 infection from either CT [12], [13] or XRay lung images [11], [14], or in the prediction of patient mortality risk, progression to severe disease, intensive care unit admission, ventilation, intubation, or length of hospital stay [8], [15].

In particular, an effective risk prediction model would contribute to precision medicine strategies for tailoring clinical management to the needs of individual patients and thereby increasing the probability of complete recovery. It would also allow emergency departments to optimize patient flow and reduce waiting times.

A substantial amount of research has therefore been been conducted with the goal of predicting patient outcome by analysing different types of data, including clinical, laboratory, and radiological features [15]–[17]. Although promising results have been reported by several authors, a recent survey of COVID-19 prognosis/risk prediction methods [15] reported that most of them are biased due to one of two reasons. Firstly, many published studies lack clinical follow-up data, implying that the categories (labels) used for machine learning may be inaccurate, because patients may develop severe complications subsequently to the initial clinical encounter used for ML. Secondly, many studies use the last available predictor measurements from electronic health records, rather than the predictor values acquired at the time when the model is intended for use. Moreover, several methods do not include any description of the study population, or the intended use of the developed models, are not explained clearly, or are not exhaustively tested. In other cases, parameter values are arbitrarily set, or the experiments at the base of hyper-parameter setting are not robust or are not reported. These considerations lead to the conclusion that most of the presented methods are poorly described and at high risk of bias, raising concern that their predictions could be unreliable when applied in clinical practice [15].

In this article, we therefore aimed to develop a rigorous and explainable risk prediction model that avoids weaknesses mentioned above. Each of the relevant steps of our algorithm was critically designed, tested, and compared to state-of-the-art techniques from the published literature. Moreover, the dataset used to develop and test the algorithms is described in detail (see Section III), and each of the methods and parameter settings used by our approach is described and motivated. The principal aim of this study is to develop an unbiased automatic system primarily devoted to selecting the most important clinical and laboratory variables to be used for COVID-19 risk assessment. Importantly, the variables considered in the present study also include two radiological scores resulting from radiologists’ evaluation of CXRs and two “lung involvement” scores computed by one of the best performing deep neural networks aimed at COVID-19 risk diagnosis. This allows assessing the integration of a radiological score computed by humans and radiological scores computed by a deep network (see Section II.B), to assess the trustworthiness of computerized AI systems, whose disadvantage is often related to their “black box” nature.

To properly manage the missing data issue that arises from the integration of multiple sources of data for COVID-19 risk prediction, we assessed a number of imputation techniques (see Section IV), including methods that do not assume Normality of the data [18]–[20] and several other methods that have been shown to be effective [21]–[23].

Secondarily, in Section V we present a novel feature selection technique exploiting a cross-validation strategy to combine the Boruta [24]–[26] algorithm and a permutation-based feature selector embedded into Random Forests (RFs, [27]). The proposed feature selection method enables robust and stable feature selection (Section V-A1).

The selected features are then used as input to RFs [27] (see Section V-A2) and to the derived Associative Trees (AT, [28], [29], see Section V-A3). While RFs produce a great number of rules, sometimes difficult to be understood, ATs are constructed from RFs, to essentially summarize them producing a simpler rule set that can easily be evaluated and interpreted by clinicians.

RFs and ATs were chosen since their interpretability does not require any (post hoc, proxy) explainer model to analyze their predictions, therefore avoiding unreliable and misleading explanations [30]. Results computed by these algorithms were compared to those obtained by Generalized Linear Models (GLM [31], Section V-B), which assume a binomial distribution for the response variable, therefore removing the normality hypothesis, and estimate a linear regression model “linked” to the response variable through a logit distribution. To avoid any bias [15], since the rules are intended to be used at the time of admission of patients to the Emergency Department (ED), our dataset is composed of data acquired upon ED admission of 300 patients, for whom five-months of follow-up data are available, and whose CXR was evaluated by two experienced radiologists blinded to patient status.

In sum, the main contributions and novelties of this article are:

• A machine learning-based computational system that can be easily deployed and used in emergency departments for an early and fast assessment of risk prediction in COVID-19 patients.
• Integration of clinical, laboratory and radiological data for the prediction of COVID-19 disease risk. The integrated prediction system includes radiological scores estimated by both expert radiologists and by specialized state-of-the-art deep neural networks.
• A novel, robust feature selection algorithm that combines the Boruta algorithm [24], [25] with permutation-based feature selection methods embedded in RFs [27], [32], [33] to select variables that are most relevant for COVID-19 risk prediction.
• An explainable machine learning decision system based on Additive Trees that can support physicians in the early COVID-19 risk assessment through a set of simple and human-interpretable decision rules.
• A thorough comparative evaluation of different imputation techniques to manage the problem of missing data in the context of outcome prediction for COVID-19 patients.

This retrospective study was approved by the ethics committee of the hospital where data were collected, which also waived the requirement for informed patient consent because of its retrospective nature.

## II. Related Work

In this section we overview related works concerning: missing data imputation methods (Section II-A) underlying the algorithms we have studied and compared in Section IV, deep learning models for diagnosis of COVID-19 from lung CT or chest CXR images (Section II-B), which are related to the deep model we use to compute automatic COVID-19 severity scores from CXR images (Section III-B), and risk-prediction methodologies (Section II-C) linked to the proposed risk-prediction models (Section V).

### A. Missing Data Imputation

Medical/clinical research is often performed on datasets with a limited number of samples, some of which are described by vectors containing missing values, and where the missing data can be described by one of the following mechanisms [18], [20], [34], [35]:

• Missing-Completely-At-Random (MCAR), meaning that the event of a value being missing is independent from both observed and missing values, and occurs totally at random;
• Missing-At-Random (MAR), occurring when the probability of missing values only depends on observed data, i.e., the latter define groups within which the probability of being missing is constant;
• Missing-Not-At-Random (MNAR), taking place when data are not MCAR or MAR, and missingness depends on unobserved data. In other words, there is a well-defined (even though often unknown) cause for missing values. In the case of MNAR, having a missing data in one variable often has some relationship with the observations of other variables. For example, values for variable x1 may be missing/observed when variable x2 has high/low values. Alternatively, values in variable x1 may be missing when values in variable x2 are also missing [34].

In any case, due to the limited number of samples, removal of points with missing data is not a good option. Instead, data imputation algorithms are generally applied, which may be grouped into three categories: methods employing statistical models to essentially estimate the underlying data distribution, methods based on machine learning techniques, and methods based on hybrid combinations of the previous approaches.

Statistical methods replace missing data by estimating their underlying distribution and/or the whole data distribution. The imputed values are drawn from the estimated distribution when a random error may be added to simulate real distributions. Examples of such methods are Hidden Markov Models [36], linear regression models [37], [38], KNN-imputation [39], cold and hot-deck imputation [40], SVD-based imputation [41], or methods that explicitly estimate the underlying distribution by using, for instance, Gaussian mixture models [42]–[44]. These methods are well suited for MAR or MNAR data because they are tied to the estimation of a distribution. However, they are often based on critical parameters having a high impact on the computed values, and setting and evaluating these parameters can be quite difficult because the ground truth (the missing values themselves) is not known.

Machine-learning methods are more recent. They perform imputation by learning the data distribution from the complete samples. For example, Random Forests [19] are particularly appealing because they deal with heterogeneous data whose features can have different data types, do not need any data normalization, and produce explainable values. Other, more complex techniques, are based on neural networks. Among them, several proposals leverage auto-encoder networks [45]–[49] or encoder-decoder Convolutional Neural Networks (CNNs) [50], [51] in order to reconstruct the training samples in their decoding output. Once trained, such decoding networks are able to reconstruct the missing values in test samples.

A completely different imputation approach is used by Generative Adversarial Neural Networks (GANN) [52], which learn to generate “missing” data with the same distribution as the training set. This is done by training a “generative” network, which generates possible imputed values and proposes them to a “discriminative” network, which is trained to accept only those generated values that properly fill the missing ones according to the underlying data distribution. Neural networks may be better able to model MCAR, MAR, and MNAR data because of their inherent non-linearity, but their main disadvantage is the need of a large training set, which is often not available in case of (bio-) medical data. Moreover, neural networks are “black box” models whose predictions are difficult to explain.

Hybrid approaches have been proposed to exploit and merge the advantages of different methods. They are essentially based on the multiple imputation approach initially presented in [53], [54] (see Section IV). Multiple imputation (MI) methods, e.g., MICE [23], [35] (see Section IV), essentially produce several estimates of the missing data by techniques containing some randomness. Then, two possible approaches are used to obtain the final result: (i) the first approach processes each imputed set in the same way and then combines the computed results through statistical methods [53], [55] (see Section VI-A2); (ii) the second one combines the computed imputations through classical techniques, such as the mean of the imputed values [56] (which may not be appropriate [35]) or by exploiting machine learning methods [57] (which require a lot of training samples).

Although MI techniques are able to produce effective results, their main parameter, i.e., the number $m$ of imputations to be generated and then combined, must be carefully chosen in order to reach a low and stable between-imputation variance (see Section IV-B1). Also, the kind of data missingness (MCAR, MAR, or MNAR) that hybrid techniques are best suited for depends on the merged imputation methods.

### B. Automated COVID-19 Diagnosis From Lung Images

Since the beginning of 2020, several deep neural models have proven their effectiveness in the diagnosis of COVID-19 infection from either lung CT or CXR images [58]. Although the proposed deep neural networks were developed upon completely different architectures, and exploit different training losses and optimization algorithms, their common trait is the “Active, Incremental Learning” approach used for learning [59], [60], which is especially needed when the available datasets are limited in size and only small numbers of new cases can be acquired incrementally.

Thanks to the existence of large open datasets containing either lung CT [61] or CXR [62] images from patients with various diseases other than COVID-19 (e.g., lung cancer, pneumonia, pleural effusion, and others), the problem of COVID-19 diagnosis is commonly addressed by training well-known existing deep neural networks [62]–[64], such as ResNet [65], [66], Inception-Net [67], [68], or VGG [69], [70], on the existing, large datasets. In this way, the network is first trained on a similar task, such as lung cancer or pneumonia diagnosis. Next, the knowledge of the pre-trained network is “incremented” by applying a training phase where an augmented COVID-dataset is used [12], [71].

Importantly, considering that deep models have been highly criticized in the past for their “black-box” explanations, several deep models proposed for COVID-19 diagnosis [12], [14] include a further interpretation step, applied to motivate the computed prediction. Among the various state-of-the-art methods for interpreting the predictions of deep models [72], the mostly used are sensitivity analysis [12], [73], [74], which allows the areas of highest activation to be identified, e.g., in the first hidden layer (since this layer is often considered as the one where base textural and color/gray level features are learned). Another common approach is output back-propagation as used by algorithms such as GRAD-CAM [13], [75] or layer-wise relevance propagation [14], [76], which essentially back-propagate the activation in the output layer to understand which areas are the most relevant in the computation of the final decision.

Deep models for lung CTs and models for CXRs differ in the dimensionality of the input images (CTs are 3D images while CXRs are 2D images), meaning that deep models with 3D convolutional layers are used for processing CTs, whereas models with 2D convolutional layers are used for CXRs. On the other hand, all the methods apply a transfer learning technique and most of them start by a ResNet or an inception-Net.

The work proposed in [13] represents an exception to the above considerations, since the authors eschew the 3D processing generally applied for CT images in favor of the classical 2D processing applied for 2D (CXR) images. The 2D ResNet50 architecture process each 2D slice of the CT and the output of all the ResNet are subsequently used as input to a max pooling layer followed by a dense layer, which computes the final prediction. Another interesting example of a deep learning model for CTs applies transfer learning to ResNet architectures and creates an augmented dataset by applying the usual image transformations to both the original image and the images obtained by wavelet decomposition. More precisely, instead of augmenting the dataset by transforming only the original image, wavelet decomposition is applied and also the images obtained from wavelet decomposition up to 3 levels are added to the training set [12].

In general, deep learning models for CT data obtain higher performance than those trained on CXR data, which presumably reflects the higher sensitivity of CT for diagnosing abnormalities related to COVID-19 as compared to CXR.

Despite this initial enthusiasm for machine learning based on lung CT data, their longer acquisition time and higher costs (when compared to chest CXRs) mean that lung CT are impracticable for the early screening of patients with suspected COVID-19 in EDs, even though CT may be the preferred modality for predicting the disease progression in COVID-19 patients. To this end, a recent study presented a severity score index computed by humans from chest CT, and used it together with other inflammatory indexes and age to form a patient’s feature vector input to logistic regression classifiers [77].

A recent approach to feature selection in COVID-19 CXR data used the first convolutional layers of existing networks (e.g. AlexNet, VGGs, GoogleNet, ResNets, InceptionNets, DenseNet) as extractors of “Deep Features”. The convolutional layers were connected to a dense fully connected layer that transforms the output of the convolutional layers into a 1000 dimensional vector, whose weights are tuned via transfer learning. The 1000-dimensional outputs are then used as input to support vector machines (SVMs). The results showed that a ResNet architecture followed by SVM achieves the best performance [78].

Based on the notion that the residual layers of ResNet are the key for its success, in [11], [14] authors presented CovidNet, a tailored CNN model using residual connections, which is trained to reproduce the scores of lung involvement (extent and severity, cf. Section III-B) produced by human experts. Given the successful results obtained by such network, we used it to produce two radiological features, which have been added to our dataset.

### C. Risk Prediction Models for COVID-19 Patients

A recent exhaustive survey of the literature on multivariate models and scoring systems for predicting COVID-19 related outcomes revealed 107 studies describing 145 prediction models. Of these, four models aim to identify people at risk in the general population; 60 exploit medical imaging for diagnosing COVID-19 in patients with suspected infection; nine models diagnose disease severity; and 50 propose prognostic models for predicting mortality risk, progression to severe disease, intensive care unit admission, ventilation, intubation, or length of hospital stay.

Besides being a precise report of all the available state-of-the-art works (up to May 5^th^, 2020) for COVID-19-related predictions based on patient data, the method proposed in [15] is very interesting since it highlights all the biases mentioned in Section I and that affect several of the published methods. However, the work in [15] does not describe the different machine learning or statistical approaches used for prediction.

In this work, we sought to update the survey of COVID-19 methods with papers published up to October 7^th^, 2020. We considered all prognosis prediction models for COVID-19 patients, in order to identify their main processing steps. First, we noted that most of the proposed approaches avoid, or do not even mention, any pre-processing phase for data normalization/standardization, missing value imputation, or feature selection, which would surely increase robustness and improve performances. Moreover, while some works only report descriptive statistics obtained by univariate [79] or multivariate [80] analysis, the majority of the approaches exploit logistic regression classifiers [81]–[97]. The remaining methods use RF classifiers [85], [87], [91], [92], [97]–[101] or XGBoost [102], [103], SVMs [87], [91], [97], [100], [101], K-Nearest Neighbor classifiers [87], [91], [100], Cox regression models [104], [105], or artificial neural networks [106].

Unfortunately, except for an approach that was developed and tested based on a private dataset with 929 COVID-19 patients [107], all the published methods were developed with datasets with relatively small sample sizes. This hinders the usage of sophisticated learning models, such as neural networks, which could uncover highly nonlinear relationships. Moreover, since all the datasets are private, an objective comparison between different methods is impossible.

## III. COVID-19 Dataset

In this section we describe our patient dataset and provide a description of the radiological feature computation used by our method.

### A. Patient Dataset

This study was performed on clinical, comorbidity, laboratory, and antero-posterior (A-P) or posterior-anterior (P-A) CXR data from patients referred to the ED of an urban multicenter health system, from March, 7, 2020, to April, 10, 2020. All patients in our cohort were RT-PCR positive for COVID-19.

Our inclusion criteria stipulated the availability of five-months of clinical follow-up data, to allow a truthful and reliable risk classification. Additionally, patients were included only if a CXR was performed and evaluated by two experienced radiologists before the availability of the nasopharyngeal swab result.

The five-month follow-up allowed us to accurately classify low-risk patients, who were either not hospitalized or, despite hospitalization, were never intubated and survived with no serious consequences, and patients at high risk, that is patients that either were intubated, experienced serious consequences, or died.

With this setting, the patient set included, 207 and 94 adult men and women with a mean age of 61 ± 1 years [min = 23, max = 95], and with a number of days with symptoms from COVID-19 that were on average 7 ± 0 [min = 1, max = 30]. Among them 214 patients were at low risk, while 87 patients were at high risk.

The data included symptoms (e.g., fever, cough, dyspnea, etc.), clinical history and comorbidities (such as arterial hypertension, chronic obstructive pulmonary disease, cancer, asthma, etc.), laboratory measurements (e.g., LDH, white blood cell count, lymphocyte), saturation/oxygen values, and patient data (age, sex).

Although effective data imputation techniques were applied to fill missing values (see Section IV), two laboratory variables lacking more than 50% of observations (LDH, AST) were removed. Moreover, to avoid singularity, variables having a variance below 0.025 were removed (precisely, logical variables recording the presence/absence of two symptoms, ageusia/anosmia and thoracic pain, and three variables recording comorbidities, that is pulmonary interstitial disease, hepatopathy, and dementia).

The resulting dataset (summarized in Table 1) is composed of 41 variables, whose values were recorded during patients visits at the ED:

• twelve are logical variables representing the presence/ absence of a symptom,
• nine are logical variables describing the presence/ absence of a comorbidity,
• patient sex is represented with a logical variable (true for men and false for women),
• four integer variables report: patient age, the number of comorbidities, the number of symptoms, and the number of symptomatic days before presentation to the ED,
• two real-valued and two integer-valued variables encode radiological features,
• two integer variables record saturation values,
• nine real values variables describe laboratory (blood) test results.

**TABLE 1:** Variables in the Patient Dataset

Variable name	All sample	Moderate risk	Severe risk	≈ p-value (chi squared test)	
Boolean variables				
Sex	207 men	145 men	62 men	0.5702
	94 women	69 women	25 women	
Symptoms	% presence (no.)			
Fever	93 (280)	92.5 (198)	94.3 (82)	0.6382
Cough	66.8 (201)	68.7 (147)	62.1 (54)	0.2804
Dyspnea	55.1 (166)	47.7 (102)	73.6 (64)	5E-04
Respiratory Failure (IR)	13 (39)	8.9 (19)	23 (20)	0.002
Myalgias	9.3 (28)	9.3 (20)	9.2 (8)	1
Other	9.6 (29)	9.8 (21)	9.2 (8)	1
Syncope	4.3 (13)	5.1 (11)	2.3 (2)	0.3598
Asthenia	12.3 (37)	11.7 (25)	13.8 (12)	0.6932
Vomiting.Nausea	5 (15)	4.2 (9)	6.9 (6)	0.3963
Diarrhea	10.3 (31)	10.7 (23)	9.2 (8)	0.8356
Headache	3 (9)	3.3 (7)	2.3 (2)	0.7346
Pharyngeal.pain	3 (9)	3.7 (8)	1.1 (1)	0.2894
Comorbidities	% presence (no.)			
Pneumo.asthma	4.7 (14)	5.1 (11)	3.4 (3)	0.5852
Pneumo.BPCO	5.3 (16)	4.2 (9)	8 (7)	0.2659
Neoplasia (last 5 years)	10.6 (32)	7.9 (17)	17.2 (15)	0.0235
Smoke	5.3 (16)	5.6 (12)	4.6 (4)	0.7836
Arterial.hypertension	29.9 (90)	26.2 (56)	39.1 (34)	0.039
Cardiovascular pathologies	16.6 (50)	11.7 (25)	28.7 (25)	0.001
Diabetes	15.9 (48)	12.1 (26)	25.3 (22)	0.0045
Obesity	6 (18)	5.6 (12)	6.9 (6)	0.7976
Celebral Stroke	4 (12)	3.7 (8)	4.6 (4)	0.7536
Integer- and real-valued variables				
	All sample	Moderate risk	Severe risk	≈ p-value (one-sided Wilcoxon signed-rank test)
Counts	mean ± s.e. [range]			
No.Symptoms	3 ± 0.09 [0, 7]	3 ± 0.1 [0, 7]	3 ± 0.17 [0, 6]	class 0< class 1, 0.0224
No.Comorbidities	1 ± 0.09 [0, 6]	1 ± 0.1 [0, 5]	1 ± 0.19 [0, 6]	class 0< class 1, 4E-04
Symptoms.No.days	7 ± 0.29 [1, 30]	7 ± 0.36 [1, 30]	7 ± 0.51 [1, 20]	class 0<class 1, 0.4752
Age	62 ± 1.15 [23, 95]	58 ± 1.34[23, 92]	67 ± 1.88 [23, 95]	class 0< class 1, 0
Radiological variables	mean ± s.e. [range]			
usa.radio.score.MAX^1^	3 ± 0.14 [0, 6]	3 ± 0.16 [0, 6]	4 ± 0.24 [0, 6]	class 0< class 1, 1E-04
radio.SCORE^1^	9 ± 0.26 [0, 18]	8 ± 0.31 [0, 16]	10 ± 0.46 [0, 18]	class 0< class 1, 0
GEO.extent.score^2^	4.22 ± 0.07 [1.45, 6.30]	4.01 ± 0.08 [1.45, 6.22]	4.74 ± 0.1 [1.73, 6.30]	class 0< class 1, 0
OPC.extent.score^2^	3.1 ± 0.06 [1.17, 4.85]	2.92 ± 0.07 [1.17, 4.85]	3.55 ± 0.1 [1.24, 4.85]	class 0< class 1, 0
Saturation values	mean ± s.e. [range]			
PaO2.PF	310 ± 9.18 [40, 733]	333 ± 9.57 [61, 733]	231 ± 16.59 [40, 567]	class 0< class 1, 0
SpO2.in.FA	93 ± 0.46 [65, 100]	95 ± 0.37 [82, 100]	88 ± 1.07 [65, 98]	class 0< class 1, 0
Laboratory variables	mean ± s.e. [range]			
ALT	35 ± 3.51 [4, 486]	34 ± 3.69 [4, 378]	42.5 ± 7.96 [9, 486]	class 0< class 1, 0.0448
Platelets	199 ± 6.6 [7, 792]	196.5 ± 8 [7, 792]	205 ± 11.69 [34, 513]	class 0< class 1, 0.4784
White.blood.cells	8.45 ± 0.71 [1.65, 179.67]	7.54 ± 0.54 [2.3, 109.77]	10.66 ± 2.05 [1.65, 179.67]	class 0< class 1, 0.0041
Red.blood.cells	4.64 ± 0.04 [2.56, 7.65]	4.68 ± 0.04[2.56, 7.65]	4.53 ± 0.07 [2.86, 6.43]	class 0> class 1, 0.0266
Lymphocyte	2.49 ± 0.76 [0.11, 172.48]	2.22 ± 0.65 [0.25, 98]	3.12 ± 2.04 [0.11, 172.48]	class 0> class 1, 1E-04
perc.Lymphocyte	17.94 ± 0.72 [0.6, 96]	19.7 ± 0.84 [3.3, 85.4]	13.72 ± 1.29 [0.6, 96]	class 0> class 1, 0
CRP^3^	8.92 ± 0.46 [0.05, 34.7]	7.01 ± 0.46 [0.05, 27.85]	13.66 ± 0.96 [0.77, 34.7]	class 0< class 1, 0
Haemoglobin	13.49± 0.11 [7.16, 19.1]	13.63 ± 0.12 [7.16, 19.1]	13.14 ± 0.21 [8.6, 17.7]	class 0> class 1, 0.0124
Haematocrit	38.88 ± 0.29 [21, 64]	39.18 ± 0.34 [21, 64]	38.12 ± 0.54 [25.3, 51.1]	class 0> class 1, 0.0336

^1^ Radiological value automatically computed by human,

^2^ Radiological value automatically computed by CovidNet,

^3^ CRP=C-Reactive Protein.

Boolean variables (symptoms, comorbidities, and sex) are described through the percentage of true values in all the patient dataset (column “All samples” in table 1), in the subset of patients at moderate risk (column “Moderate Risk” in table 1), and in the subset of patients at severe risk (column “Severe Risk” in Table 1). Integer and real valued variables are represented through their mean ± standard error (s.e.) of the mean and their range ([minim value, maximum value]) in the entire dataset (column “All samples”), the subset of patients at moderate risk (column “Moderate risk”), and the subset of patients at severe risk (column “Severe Risk”).

To provide a first hint of the class separation provided by each variable, we performed statistical analysis to check whether there are statistically significant differences in the patients distributions. Precisely, for boolean variables we applied the chi-squared test to determine if statistically significant differences were present between patients at low or at high risk. Numerical variables were analyzed to detect statistically significant distribution differences by applying the one-sided Wilcoxon signed-rank test.

### B. Chest X-Ray Analysis and Automated Processing

The Fleischner Society presented three different scenarios and an algorithm for recommending chest imaging that includes CT and/or CXR to direct patient management during the COVID-19 pandemic. Ultimately, the choice of imaging modality is left to the judgement of clinical teams at the point of care, accounting for the differing attributes of CXR and CT, local resources, and expertise [2]. Though CXR shows clear patterns, distinguishable from those of pneumonia [108], when COVID-19 infection becomes serious, it is insensitive in mild or early infection stages [108]. In contrast, lung CT has greater sensitivity for early pneumonic changes, but this advantage is partially diminished by the huge burden placed on radiology departments in terms of staff commitment, CT room workflow, and disinfection procedures [2], [109]. Therefore, many Italian hospitals decided to employ CXR as a first-line triage tool [108]–[111].

Several recent studies on the utility of initial CXR for predicting clinical outcome correlated the presence and the extension of opacities on initial CXR with the need for hospitalization and/or intubation [17], [108], [110], [111].

In light of these considerations, in our study we included four radiological variables expressing the extent and severity of the COVID-19 pattern, visible from the CXR acquired at the time of presentation to the ED.

Two of the four radiological variables, *radio.score* and *usa.radio.score*, were evaluated by expert radiologists, which were blind to the patients’ condition; the other two radiological variables, *GEO.extent.score* and *OPC.extent.score*, were computed by a deep neural network trained on a radiological score evaluated by clinical experts [11], [14].

*Radio. score* and *usa.radio.score* were defined by two thoracic radiologists with 23 and 20 years of experience in thoracic imaging, after re-evaluation of the initial CXR that the patients underwent during the admission at the ED. The *radio.score* index was used to assess the severity of pulmonary involvement from both the 156 antero-posterior (A-P) and the 143 postero-anterior (P-A) images. The score is calculated by dividing each lung into three areas (upper, middle, and lower); each area is then scored with an involvement value in the range $\{0, \ldots, 4\}$, where 0 means that no anomaly has been found, while higher scores mean increased presence of severe COVID-19 CXR patterns: 1 = reticular interstitial thickening, 2 = reticular interstitial thickening and ground glass, 3 = ground glass opacities and consolidation with ground glass as the most widespread anomaly, 4 = consolidation as the most widespread anomaly. Summing up the scores assigned to each of the six areas, each lung gets a score in the range $\{0, \ldots, 24\}$. Lin’s concordance correlation coefficient [112] between the scores of the two radiologists ($c_{\mathrm {Lin}} = 0.76$, c.i. $= [{0.65, 0.76}]$, p-value $ < 1\text{E}$-58) showed a substantial agreement. Therefore, we averaged the two scores to get a single value.

By binarizing the scores of each lung area, that is by assigning a value of 1 to each area showing at least ground glass opacities and consolidations (area scores greater or equal to 2), an summing up all the binary values, we obtained a simplified version of *radio.score*, falling in the range $\{0, \ldots,6\}$ and referred to as *usa.radio.score*. Since in this case Lin’s correlation coefficient showed a low agreement ($c_{\mathrm {Lin}}=0.40$, $\text {c.i.} = [{0.30, 0.49}]$, p-value $ < 1\text{E}$-11), a pooled score was obtained by taking the maximum value for each patient. This is a conservative way of pooling the results, based on the assumption that a false positive error is less costly than a false negative error. In other words, diagnosing a mild case as severe is better than wrongly considering a severe case to be mild.

To assess the reliability of the scoring system computed through a deep network, we used the state-of-the-art CovidNet deep neural network [11], [14]. Precisely, we automatically preprocessed the CXR images of each patient to first remove positional artifacts, such as rotations and variations in zooming. Subsequently, gray levels were normalized through ACE [113], a spatial color equalization algorithm [114] that has been often used to remove unwanted and adverse illumination conditions [115] and that recently gained importance in the field of medical image processing [116], thanks to its ability to reveal small details without introducing noise and artifacts. The preprocessed images of each patient were used as input to CovidNet in order to get a geographic extent score (*GEO.extent.score*) and an opacity extent score (*OPC.extent.score*).

CovidNet is a deep neural network that was originally developed for recognition of COVID-19 patients [14]. CovidNet was subsequently extended by transfer learning on an augmented dataset composed of only 130 CXRs from Chinese patients [14], which were scored by two experienced radiologists by adapting the scoring system proposed in [116]. Such scoring method quantifies both the extent of lung involvement by ground glass opacity or consolidation, through the geographic extent score (*GEO.extent.score*), and the kind of COVID-19 patterns seen in the radiographs, through the opacity extent score (*OPC.extent.score*). Both scores are computed separately on each lung, and a final value is then obtained as sum of the left- and right-lung scores. More specifically,

• *GEO. extent.score* takes the following scores on each lung: 0 = no lung involvement; 1 = < 25% of lung involvement; 2 = 25-50%; 3 = 50-75%; 4 => 75% of lung involvement; thus, the final score ranges from 0 to 8.
• *OPC. extent.score* ranges from 0 to 6, and it quantifies the degree of opacity in each lung by using the following values: 0 = no opacity; 1 = ground glass opacity; 2 = consolidation; 3 = white-out [11].

## IV. Approaches to Missing Data

The available dataset contains both logical, integer-valued, and real-valued attributes. Both the discrete and continuous variables are affected by missing data; thus, it is appropriate to consider an imputation phase.

### A. Uncovering the Missing Data Mechanism

Since the validity of any imputation method depends on the missing data mechanism, care must be taken to understand whether the involved data are MCAR, MNAR, or MAR [18], [20], [34], [35].

**FIGURE 1.**
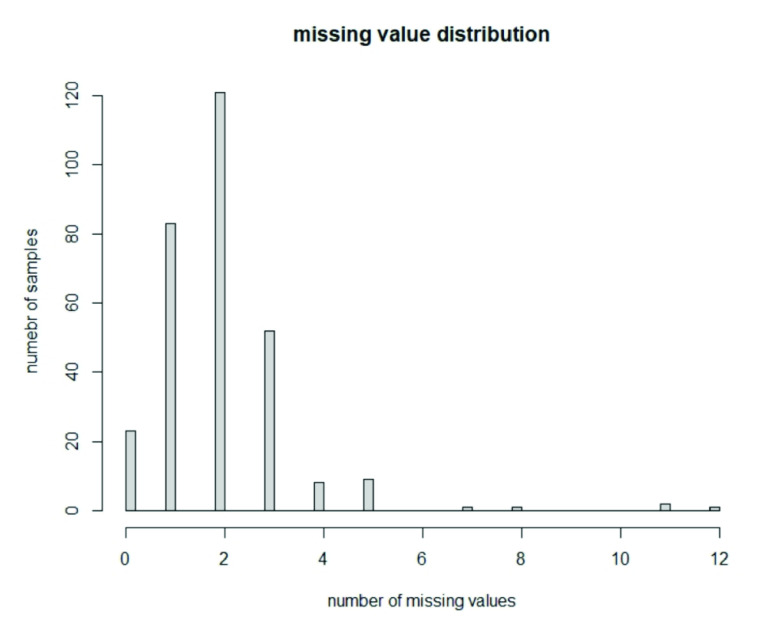
Histogram of missing values for each sample: the maximum number of missing values is 12, corresponding to 25% of the variables. Only one sample has 12 missing values.

**FIGURE 2.**
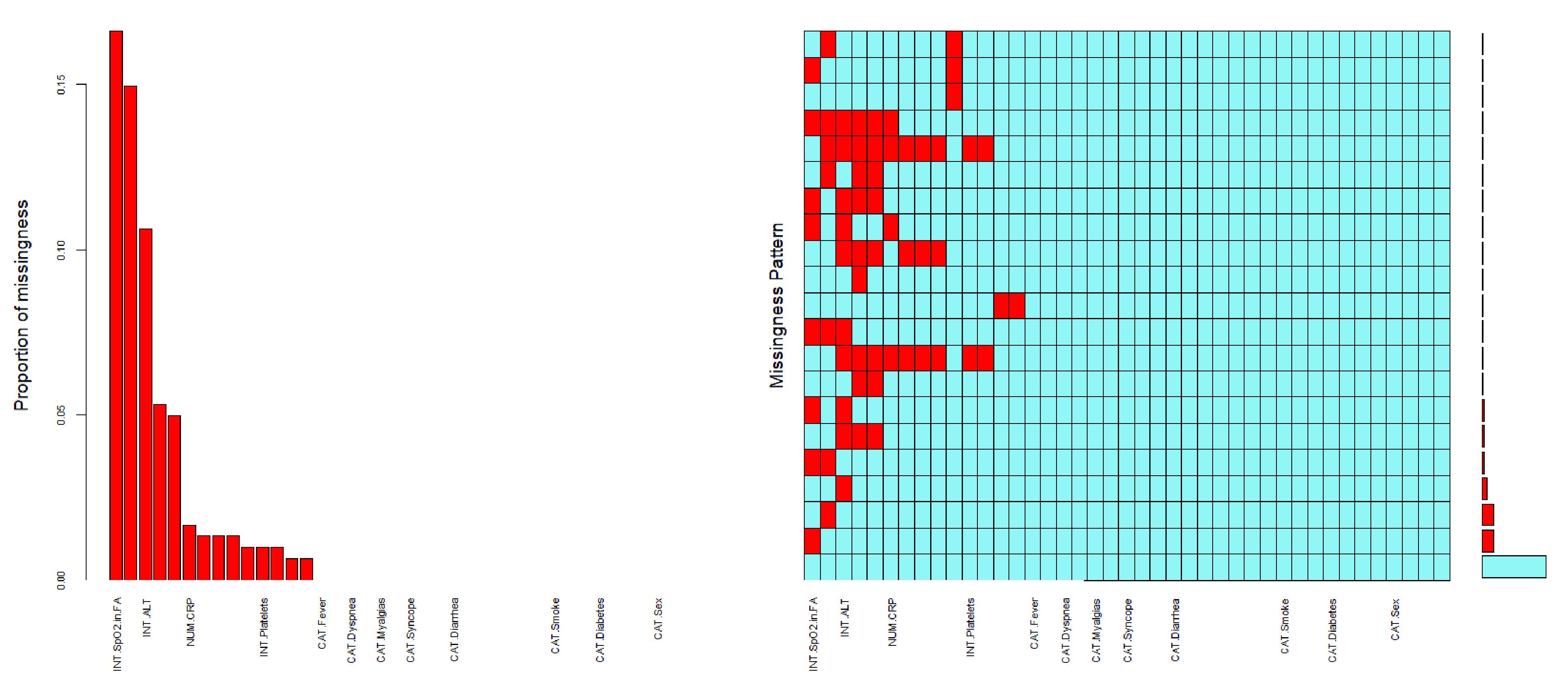
Missing data patterns. (left) Proportion of missing values for all variables in the dataset, sorted by decreasing order. (right) Combinations of missing values: red squares in a matrix entry denote the presence of missing values for the variable associated to the column in the samples corresponding to the row; the bars on the right show the cardinality of each set of points.

Precisely, to confirm that data is, or is not, MNAR, the missing data pattern is generally observed through visualizations (see Fig. 1 and Fig. 2). We searched for some MNAR pattern by expressing each attribute as a binary variable, whose observations are set to 1 (missing) or 0 (observed). Using this binary representation, we applied the following analysis, which provided no evidence of MNAR data. For each pair of attributes, we found no high correlation between the corresponding binary representations (Pearson correlation coefficient < 0.75, with a significant p-value), or we confirmed the independence of their missing/observed data proportions, using the chi-squared test (with Yates’s correction). Further, for each variable with a sufficient number of missing values (we set this value to be 25), $x_{1}$, and each other numeric variable, $x_{2}$, we used the Wilcoxon signed-rank test to confirm that the difference between the distributions of $x_{2}$ values for missing and observed values of $x_{1}$ was not statistically significant.

Next, to determine whether our data are MAR or MCAR, we used the non-parametric test of Jamshidian and Jalal [18], [20], an extension of Little’s test [117] that is suited both in case of a high and a low proportion of missing values. Precisely, if homogeneity of covariances (homoscedasticity) between subsets of data having identical missing data patterns is detected, data are supposed to be MCAR. The novelty of the approach relies on the fact that authors test for homoscedasticity using a modification of the statistic proposed by Hawkins [118]. This statistic has the peculiarity of working well for testing homoscedasticity in *complete* data when group sizes are small. In order to process a complete dataset, in case of unknown data distribution authors perform imputation by a method, *distFree*, that only assumes independence of the observations, and the continuity of their cumulative distribution function; no further specific distributional assumptions are required. *distFree* is similar to the imputation technique proposed in [119], which exploits maximum likelihood estimators to compute a linear predictor of the missing observations, and then adds a random error to obtain the final imputations. Although this method implicitly assumes that the variables are linearly related, authors argue that the maximum likelihood technique may indeed provide consistent estimators [120]. In sum, using Jamshidian’s and Jalal’s test we determined that our data are MCAR.

### B. Missing Data Imputation

At the state of the art, several imputation models for MCAR methods have been presented that can deal with “complex” data [45]. Among such methods, we experimented both Multiple Imputations by Chained Equations (MICE [23], [121]), using either predictive mean matching (*micePMM*) or Random Forest classifiers (*miceRF*) as the base imputation model, and *missForest* [19], which also exploits RFs.

More precisely, MI techniques [22] are an effective strategy that exploit randomness for producing unbiased estimates, with a reduced dependency on the normality assumption [22]. MIs are mainly used for estimating the linear or logistic regression coefficients that link predictor variables to a response variable. In this case, given a dataset (with MCAR or MAR values) and an imputation model containing some randomness, $m$ imputed datasets are drawn, and subsequently processed separately but identically by the chosen estimator. The resulting coefficients are expressed through their mean and (global) variance, computed according to Rubin’s Rule [53], [55] (see Section VI-A2), which in turn allows the Wald test to be applied for checking their significance [122]. Note that, although some authors [123], [124] suggest that setting $m=5$ MIs is enough to produce unbiased estimates, other contributions [35], [125] show that $m > 20$ should be used to obtain reliable estimates for the global variances, so that the simulation error is almost cancelled (in Section IV-B1 we experimentally determine a value for $m$ minimizing the variance).

MICE (aka Fully Conditional Specification, or FCS) is a MI technique that uses a set of conditional densities for each variable with missing data to build a multivariate imputation model on a variable-by-variable basis. Initially, all missing values are replaced by simple random sampling with replacement from the observed values. Subsequently, when using predictive mean matching (PMM [126]) as the base imputation model, the following steps are applied:

• starting from the first variable, $x_{1}$, a regression model is fit to the observed x1 by using the remaining variables as the independent predictors;
• randomness is introduced by drawing a subset of regression coefficients from the posterior predictive distribution of the computed coefficients; the drawn coefficients are used to predict all (observed and missing) values for $x_{1}$;
• each missing value in $x_{1}$ is finally imputed by considering the predicted value of one among $k$ donors, randomly selected among observed elements in $x_{1}$ whose predicted values are close to the predicted value for the case with missing data.

This process is repeated by using all the variables as independent predictors. When all variables are imputed, a cycle is complete. To stabilize the process, the cycle is repeated $n$ times by using, at each iteration, the previously imputed values as initialization values (authors suggest setting $n$ in the range $\{10, \ldots, 20\}$ for obtaining unbiased results [126]). Note that the variable order used by the iterative univariate imputation may be defined according to different criteria based on missing value proportion, such as decreasing, increasing, or random sorting.

As highlighted in [35], PMM has the advantage of using an implicit data model, thus avoiding the explicit definition of the distribution of missing values, which often brings to model misspecification. Moreover, the values imputed by PMM are actually observed values, therefore avoiding the generation of out-of-range imputations. However, PMM-based MICE (*micePMM*) is a parametric approach that assumes that the observed data have a distribution similar to that of missing data [127]. To avoid any parametric approach, a novel model was presented (*miceRF*), where RFs substitute PMM. More precisely, for each variable, a bootstrap sample is used to impute missing values in the dependent variable by using RFs. The advantage lies here in the usage of a further internal bootstrap sampling, allowing each tree to be fit to a different data sample. Results aggregated by the RF are therefore supported by a source of randomness that is greater than that of PMM; moreover, RFs do not rely on any specific assumption regarding the distribution underlying missing data. Indeed, results shown in [127] suggest that both *miceRF* and the *missForest* algorithm produce more robust results than those computed by *micePMM*.

*missForest* [19] iteratively exploits the ability of RF classifiers to deal with mixed data types without making any assumption about the underlying data distribution. It follows an iterative approach similar to that applied by MICE, that is it iteratively imputes each variable with missing data using the remaining variables. After making an initial guess for the missing values, e.g., by using the mean of observed values, it considers in turn each variable $x$ with missing entries (by default, variables are considered by increasing missing value proportion, though other sorting criteria can be used). An RF is fit to the observed values of $x$ using the other variables as predictors, and subsequently used to impute the missing values. Such procedure is repeated until the difference between the newly imputed data matrix and the previous one increases for the first time with respect to both continuous and categorical variables (obviously using two separate difference metrics). *missForest* has the same appealing properties of *micePMM* and *miceRF*. Indeed, since RFs are trained on bootstrapped samples, MIs can be computed by using different bootstrap sets, which introduces randomness. Moreover, this method can deal with multivariate data consisting of continuous and categorical variables. Finally, *missForest* does not require assumptions about distributional aspects of the data, nor does it have critical hyper-parameters to be tuned. More precisely, it only requires the number of trees ($n_{t}$) to be specified; although this value is generally set to a high value, e.g. $n_{t} = 500$, we set $n_{t} = 100$ to avoid overfitting and reduce computational time.

The first aim of this work is to provide suggestions about the employment of imputation techniques using different baseline theories. Therefore, we experimented with *distFree*, *micePMM*, *miceRF*, and *missForest*, considering both univariate imputation orders defined by the increasing and decreasing missing values proportion (henceforth referred to as “increasing imputation” and “decreasing imputation”, respectively). To avoid bias, all imputations were performed after discarding the point labels.

Note that, though *distFree* and *missForest* are not MI techniques, they both rely on a randomness source (*distFree* adds random noise to each imputation, while *missForest* trains RFs by using randomly bootstrapped samples) and may be therefore used to produce ${m}$ different imputations. In all imputation algorithms we set the maximum number of iterations to 11, since values in $\{10, \ldots, 20\}$ allow unbiased imputations to be obtained [126]. Finally, we limited *miceRF* and *missForest* univariate imputations by training RFs with a maximum of $n_{t} = 100$ trees.

#### 1) Choosing the Proper Imputation Algorithms and the Value for $m$

To compare the imputation algorithms and the univariate imputation order we produce $m = 100$ different imputations and analyze the between-imputation variance. Given the original dataset $D$ with $S$ missing values, $x_{\mathrm {miss}}(s)$ ($s = 1, {\dots }, S$) and given an imputation method, imp, specified by an imputation algorithm and an univariate imputation order, let’s denote with $D_{\mathrm {imp}}(1), {\dots }, D_{\mathrm {imp}}(m)$ the $m$ imputations produced with imp.

To compute the between-imputation variance, we found the normalization coefficients that allow the observed values in each column to be mapped to the range [0, 1] (they depend on the minimum and maximum of the observed values in each column of $D$) and used them to normalize each imputed set, therefore obtaining $D^{*}_{\mathrm {imp}}(i)$ ($i = 1, \ldots, m$). Next, we computed the between-imputation variance of each missing value $x_{\mathrm {miss}}(n)$ in $D$, $\mathrm {Var}(x_{\mathrm {miss}}(s))$, $s = 1,\ldots,S$, by using its $m$ imputed values in $D_{\mathrm {imp}}(1), {\dots }, D_{\mathrm {imp}}(m)$. The global between-imputation variance was finally computed as the mean of the $\mathrm {Var}(x_{\mathrm {miss}}(s))$, $s = 1, {\dots }, S$.

**FIGURE 3.**
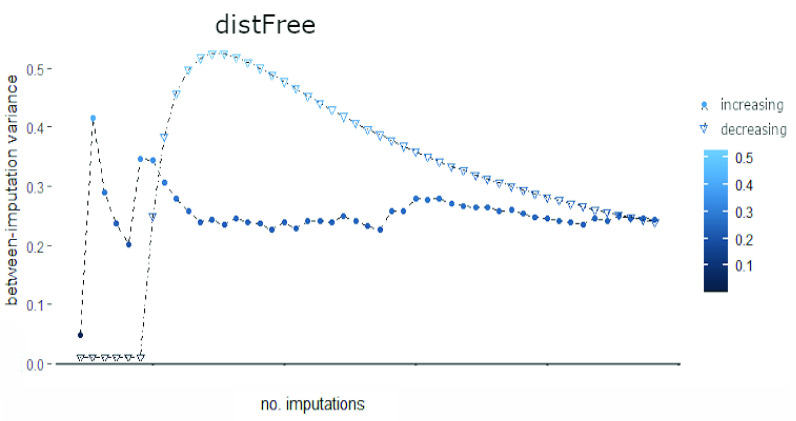
Between-imputation variances computed on 100 datasets imputed with *distFree*. Dots and triangles mark the variances computed using increasing and decreasing imputation order, respectively.

**FIGURE 4.**
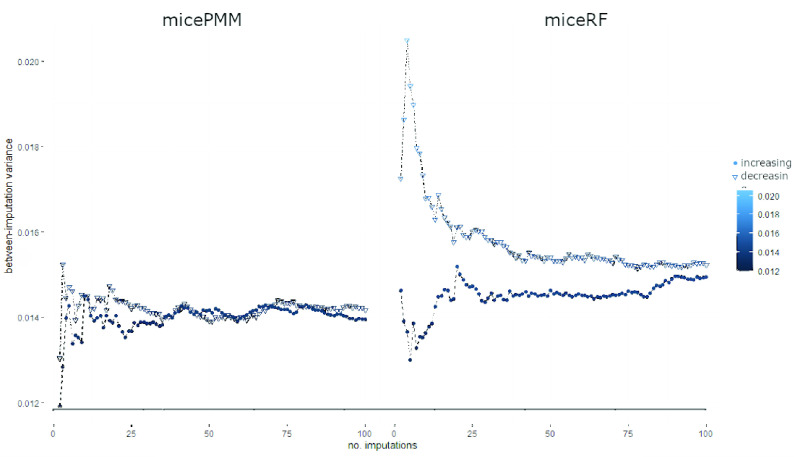
Between-imputation variances computed on the 100 datasets imputed with *micePMM* (left) and *miceRF* (right), using the same scale for Y axis. Same notations as in Fig. 3.

**FIGURE 5.**
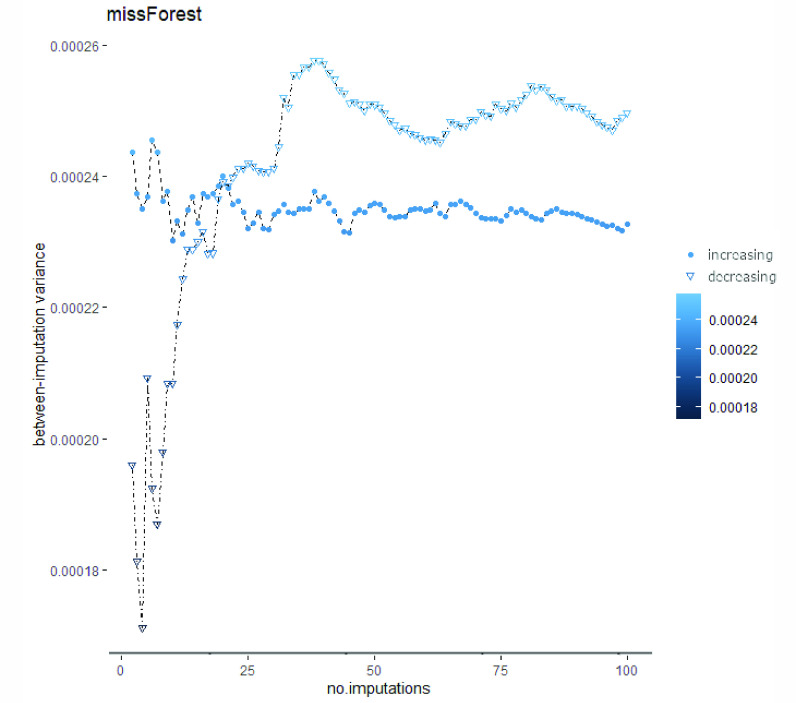
Between-imputation variances computed on the 100 datasets imputed with *missForest*. The obtained values are negligible, as highlighted by the span of Y axis: this practically means that the imputed values are always similar. Same notations as in Fig. 3.

Figs. 3–5 show the global between-imputation variances achieved by the four methods (using the increasing (dots) and decreasing (triangles) order of missing values) for $m \in \{2, {\dots }, 100\}$. In Table 3 (Appendix A) the ranges of such between-imputation variances are reported.

For both univariate imputation orders, *distFree* achieves the highest between-imputation variances with a mean slightly lower than 0.3 (Fig. 3; cf. also Table 3 in Appendix A); this variance is very high, considering that data are normalized. Moreover, the between-variance ranges of *distFree* are respectively 10^3^ and 10^2^ times bigger than those of *missForest* and of multiple imputations exploiting MICE. The high between-imputation variance computed when using *distFree* practically means that, for each missing value, its imputed values are very noisy. On the contrary, *missForest* has negligible between-imputation variance, meaning that the predicted values are stable.

Each imputation method is characterized by a between-imputation variance whose order of magnitude is independent of the univariate imputation order. However, *distFree* has completely different behaviors when the two orders are used, further suggesting that its results may not be considered as sufficiently robust. In Fig. 4 we show the zoomed between-imputation variances achieved by *micePMM* and *miceRF*, while those computed when using *missForest* are plotted in Fig. 5. When using such algorithms, the between-imputation variances reach a sort of *plateau* after an initial variability involving around 30 imputations. Although the plot based on *missForest* suggests a higher variation of the between-imputation variance, all the values are near to zero. In sum, when using the increasing univariate imputation order, both *micePMM* and *missForest* obtain the lowest between-imputation variance.

Note also that the negligible between-imputation variances produced by *missForest* highlight the fact that the different imputations it computes are very similar. For this reason, this method should not be used to impute missing data when there is the need to test the robustness of subsequent processing steps w.r.t. data variability. On the other hand, *missForest* should be used when the goal is to obtain (almost) reproducible results.

When the underlying data distribution is unknown, we therefore suggest performing imputation with either *missForest*, when negligible between-imputation variances are needed, or *miceRF*, because it combines the advantages provided by working on multiple imputations and therefore allows the robustness of subsequent algorithms to be tested by considering some randomness in the data. Obviously, when the normality assumption holds *micePMM* is also a viable option. Moreover, to achieve stable between-imputation variances, in our problem we suggest using ${m} > 20$ imputed sets as advised in [35], [125].

In the problem under study we cannot make assumptions about the underlying data distribution; therefore, though *micePMM* achieves low and stable variances, its use would not guarantee a proper imputation of missing values. Anyhow, *miceRF* has similar variances and therefore we can use it to assess MICE-based techniques, comparing it to *missForest*, which obtained the lowest between-imputation variance. With both methods we choose to use the increasing univariate imputation order, which produces more stable results, generating 50 imputed sets. More precisely, after imputing missing data by using these methods, we trained and tested RFs, ATs, and GLMs (see Section V) in order to obtain predicted risk levels, as well as the relevance of each variable in the prediction. For each method, the predictions and relevance computed on the $m$ imputations were pooled by applying Rubin’s rule (see Section VI-A2).

## V. Risk Prediction Approaches

Once missing data have been imputed, we apply two different risk prediction approaches, both described in this section.

Given a training set, the first approach firstly applies a feature selection algorithm,^1^ which combines the Boruta algorithm [24], [25] and permutation-based feature selection methods embedded in RFs [27] through a cross-validation strategy (see Section V-A). Secondly, RF classifiers (Section V-A2) are trained on the selected features. To summarize and “explain” the trained RFs, ATs [28], [29] are generated by the former trained RFs (Section V-A3).^1^Feature selection is applied on the training set to avoid incurring a selection bias [128].

The results computed by RFs are then compared to those obtained by applying GLMs [31], [129] (see Section V-B). GLMs have been chosen since they may be considered as a more powerful extension of logistic regression models, which have been widely used in the medical research field both for their simplicity and for the explainability of their predictions. Since GLMs use a combination of Lasso and Ridge constraints to select the most important features, they were applied to the imputed set without previously applying any feature selection algorithm.

### A. Feature Selection and Risk Prediction With Boruta, Random Forest and Associative Trees

In this section we describe the overall induction process at the basis of the proposed risk-prediction scheme exploiting RFs and ATs.

#### 1) Feature Selection

Feature selection is performed on the training set through an internal 5-fold cross-validation (5-cv), where 4 folds are used in each iteration as an “internal” training set to train a RF classifier on the features selected as confirmed or tentative by the Boruta algorithm.

Precisely, Boruta [24]–[26] starts with the complete set of features and applies $n$ iterations that each train a RF on a feature set augmented by “fake features” obtained by random permutations of the actual ones. The features that, for a statistically significant number of iterations, are less/more relevant than all the fake features (relevance is quantified by the mean decrease in accuracy when the feature is permuted), are selected and removed/confirmed. When Boruta has executed $n$ iterations, all features for which a decision has not been taken are returned as tentative. Boruta is a promising feature selection method whose analysis of shuffled, fake features mitigates the impact of false correlations between features and target labels, which sometimes leads to overfitting [25]. However, even when setting a high value for $n$, some features are returned as tentative. Unfortunately, the relevance computed by Boruta cannot be used for selecting/discarding such features because such value is biased by the fake features used by the method. Moreover, Boruta does not account for class imbalance. To remove some uncertainty, Boruta is therefore also internally applied within a 5-fold cross-validation (5-cv), and all the features returned as confirmed are selected, together with those selected as tentative at least 3 out of 5 times.

The existence of tentative features and the lack of robustness with respect to class imbalance is the reason why we applied the 5-cv, which trains weighted RFs on the confirmed and tentative features: this approach assigns a “permutation test importance” [27], [32], [33] to each of the features, in turn evaluated on the left out fold. Therefore, after the 5-cv iterations, the mean importance for each feature is computed and normalized so that the sum of the normalized importances equals one. The most important features are finally selected by sorting the normalized importance in decreasing order and selecting the features that retain 0.95 of the cumulative sum.

The feature importance can be evaluated by using either the “mean decrease in node impurity” (via the Gini criterion), which essentially evaluates how much each feature decreases the mean impurity over all the trees of the forest, or the “mean decrease in accuracy” after feature value permutation, which essentially evaluates how the accuracy of the prediction over the training set decreases when the feature values are shuffled. We preferred the “mean decrease in accuracy” (also called “permutation test”) to the Gini criterion, since the latter may lead to biased results [32], [33].

Once the most informative features have been selected, the selected feature set is input to RFs (described in the next subsection V-A2), which are trained to predict the patients’ risk. Subsequently, the trained RFs are merged to summarize all their rules through Associative Decision Trees (subsection V-A3), which provides more explainable predictions.

#### 2) Risk Prediction Trough Random Forests

The main advantages of RF classifiers are the potential explainability of their decisions, their capability of computing adimensional importance measures (“mean decrease in accuracy”) describing the relevance of each variable in the risk prediction task, and the few number of involved hyper-parameters [27]. The main hyper-parameters of RFs are:

• the number of trees to grow: this parameter was set to 100 since grid search allowed us to discover that higher numbers of trees not only increase computational time, but also tend to produce overfitting
• the number of variables to sample for each split: this number is automatically set in order to maximize the misclassification cost on the training set, by a greedy search algorithm which evaluates all the points in the range $\{ n_{\mathrm {feat}}/3, {\dots }, n_{\mathrm {feat}} \}$, where $n_{\mathrm {feat}}$ is the number of features obtained after feature selection;
• minimum size of terminal nodes, where the size of a node is the number of training samples falling in that node: low values for this parameter may cause overfitting and tend to grow tall trees; based on this consideration and following the advice of clinical experts, we require that the minimum node size is 10.

Though easy to use, RFs are not robust with respect to class imbalance. Therefore, training was performed by constraining the number of bootstrapped samples per class to be less than or equal to the number of samples of the underrepresented class [130]. Moreover, recalling that RFs are trained and tested by applying a 10-cv, at the end of the latter we have 10 importance measures for each variable. To obtain a single estimate for each variable, we first normalize the importance computed in each cross-validation run so that they sum to one; the global estimate of each variable in the 10-cv run is then computed as the average of the normalized importance for that same variable.

#### 3) Associative Trees Generated by Random Forests

**FIGURE 6.**
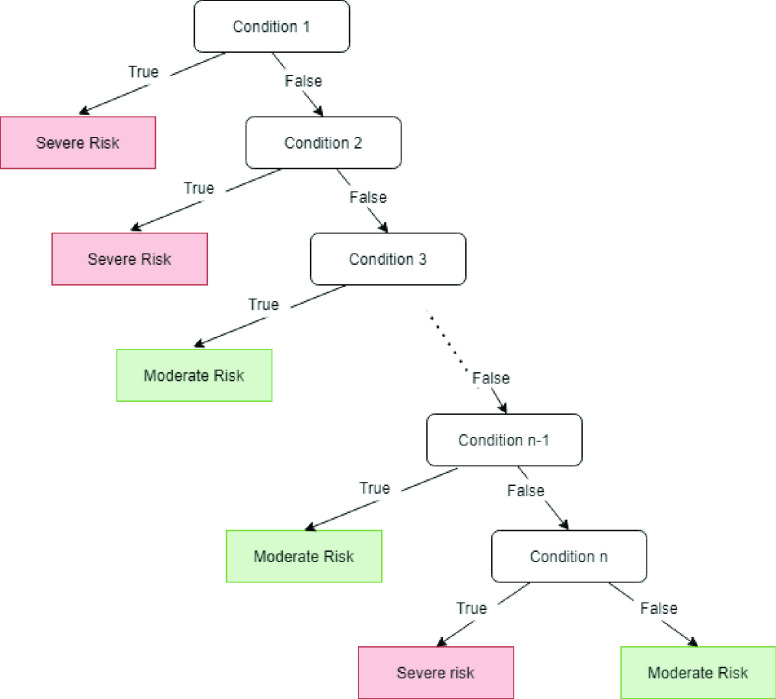
An associative tree. The tree consecutively evaluates all the conditions, until a condition is met, bringing to a decision.

As mentioned before, RFs are considered as relatively explainable models since their output is a set of decision trees, each describing a set of classification rules. When a novel sample must be classified, all the trees in the trained RF provide their response and majority voting is used to provide the pooled response. Despite the simplicity of this process, retrieving the rules that led to a specific classification becomes difficult when many trees are grown. For this reason, we translated each trained classifier into a simple associative tree, as described in [28], [29]. Associative classifiers are defined as models made of rules “whose right-hand side are restricted to the classification class attribute” [131]. In other words, they are composed by a set of rules which are consecutively evaluated. The first rule that is met provides the classification label. Associative Trees (ATs) are a simple representation of associative classifiers (see Fig. 6), characterized by the fact that each node which is not a terminal node has one child which is a leaf node.

To generate an AT from a trained RF, the following steps are consecutively applied.

1) All trees are translated into logical expressions, through a process that follows the paths from roots to leaves. Since the most informative splits often occur in the top level of a tree, the rule extraction process is stopped when it reaches the node at depth 6 [28], [29]. This procedure allows a rule to be extracted from each tree that is composed of a maximum of 6 atomic conditions joined by the logical AND operator. Each atomic rule is expressed as ${C \implies T}$, where $C$, referred to as the condition of the rule, is a conjunction of variable-value pairs, and $T$ is the outcome of the rule.
2) The trees resulting from RFs are sometimes redundant; the first step after the rule extraction is therefore aimed at applying logical simplification to the rules, discarding redundant duplicates.
3) Next, each rule is pruned by eliminating its atomic conditions whose removal increases the classification error by not more than 0.05. The error of a rule is intended as the proportion of misclassified instances among all those satisfying the rule condition.
4) After pruning, each rule is expressed by a binary vector, whose length is equal to the number of samples. Each element of the vector is set to one if and only if the rule is satisfied for the corresponding sample. This encoding is used in order to apply a simple feature selection algorithm [29], which in turn allows discarding redundant and non-informative rules. A further reduction is done by discarding rules whose frequency is less than 0.01, where frequency is defined as the proportion of training instances satisfying the rule condition.
5) The remaining set of rules is finally used to combine an AT, by using a greedy iterative algorithm; at each iteration, the best rule (intended as that with lowest error, breaking ties by taking the most frequent rule) is added to the tree until no more rules remain. After inserting each best rule, all remaining rules are re-evaluated and those with a frequency lower than 0.01 are removed before the iteration continues.

### B. Generalized Linear Models

GLMs [31], [129] generalize linear regression by allowing the learnt linear model to be related to the response variable via a link function. Ordinary linear regression estimates the coefficients of a linear model combining a set of variables for predicting the expected value of the response variable, implying normality for the conditional distribution of the response variable given the values of the explanatory variables in the model. GLMs allow this conditional distribution follow different models, e.g., Gaussian for continuous responses or binomial when dealing with a binary response.

In our problem, the binomial function links the linear combination of explanatory values to the response variable; in practice, given a training set $T=\left \{{x_{1},\,\,\ldots,\,\,x_{N}}\right \}\subseteq \mathbb {R}^{p}$ containing $N$ samples and their labels $\left \{{y_{1},\ldots,~y_{N}}\right \}~\in [{0,1}]$, GLMs find the $p+1$ coefficients $(\beta _{0},\,\,\beta)\,\,\in \mathbb {R}^{p+1}$ by using a penalized logistic regression, whose objective function uses the negative binomial log-likelihood:

\begin{align*}&\min _{(\beta _{0}, \beta) \in \mathbb {R}^{p+1}}\, -\Big [\frac {1}{N} \sum _{i=1}^{N} y_{i} \cdot (\beta _{0} + x_{i}^{T} \beta) \\&\hphantom {\min _{(\beta _{0}, \beta) \in \mathbb {R}^{p+1}} -\Big [\,}-\, \log \big (1+e^{(\beta _{0}+x_{i}^{T} \beta)}\big)\Big] \\&\hphantom {\min _{(\beta _{0}, \beta) \in \mathbb {R}^{p+1}}\,}+ \,\lambda \big [(1-\alpha)||\beta ||_{2}^{2}/2 + \alpha ||\beta ||_{1}\big]\end{align*}

Note that the objective contains an (elastic-net) penalty factor, weighted by the tuning parameter $\lambda $ which not only reduces the negative effect of degeneracies when $p > N$ or $p \approx N$, but also regularizes and selects the most important variables. Such penalty factor mixes ridge constraint (when $\alpha = 0$, which tends to select correlated predictors shrinking their coefficients [132]) and lasso constraint ($\alpha =1$, which selects only one of the correlated predictors [132]).

In our implementation, GLMs work on standardized data, and grid search is applied through an internal 10-cv to automatically choose the most suitable values for $\lambda $ and $\alpha $. The coefficients computed for each variable are often regarded as an (adimensional) measure related to the importance of the variable in the prediction problem. Recalling that, for each imputed set and fold stratification we obtain an unbiased evaluation by applying 10-cv, we averaged the coefficients obtained in the 10 folds to compute a unique coefficient for each feature.

## VI. Results

Our dataset $D$ contains 41 features; 14 (numeric) features (saturation values and laboratory values) have missing values, for a total of 188 missing values. Among features with missing values, those having the highest number of missing values are the two variables related to oxygen (saturation) values (SpO2 in free air, having 50 missing values, and PaO2.PF, having 45 missing values; both values are missing for only 9 patients), followed by lymphocyte values (%lymphocyte has 16 missing values, lymphocyte count has 15 missing values, and all patients with missing lymphocyte values have also %lymphocyte missing); the other 10 features lack at most 5 values.

In this this section we firstly report the experimental setup (Section VI-A); secondly, we report an exhaustive description of the computed results (Section VI-B).

### A. Experimental Setup

To obtain an unbiased evaluation, all the risk prediction models were trained and tested on each of the $m=50$ imputed sets, by applying an (external) stratified 10-fold cross-validation (10-cv).

Further, since the results may depend on the specific randomly computed 10-fold stratification, each risk predictor model is applied on each imputed set $n_{\mathrm {cv}} = 5$ times by applying $n_{\mathrm {cv}}$ different 10-fold stratifications.

In this way, given a performance evaluation measure among those we chose to collect (described in subsection VI-A1), for each imputed set we obtain $m\times n_{\mathrm {cv}}$ values, which are combined through Rubin’s rule [22], [122], [123] (described in Section VI-A2), and statistically compared with a one-sided Wilcoxon rank-signed test (see subsection VI-A3).

#### 1) Performance Evaluation Measures

Several published methods were evaluated by the C-statistic (that is, the area under the ROC curve, or AUC [133]). The C-statistic is the probability that the model predicts a higher risk for positive samples. Moreover, it is adimensional, and thus it allows the comparison of different predictors. However, as highlighted in [133], the C-statistic is not an exhaustive description: for instance, it does not account for uneven class distributions, and hides the method performance on the positive or on the negative samples. To provide an exhaustive description, we therefore decided to record also sensitivity (performance on positive samples), specificity (performance on negative samples), accuracy (ratio of misclassified samples), and F1 score (harmonic mean of precision and recall, which accounts for uneven class distributions). In practice, we used the AUC to select the most promising combinations of imputation method, univariate variable imputation order, and risk prediction method (RFs, ATs, or GLMs). We subsequently selected the most appropriate risk prediction model by analyzing the performance on the positive and negative samples, as described by the remaining performance measures.

#### 2) Combining Results Through Rubin’s Rule

Given the imputed set, we obtain a robust comparative evaluation by training and testing each predictor model (RF, AT, or GLM) $n_{\mathrm {cv}} = 5$ times on each of the $m$ imputed sets, by using $n_{\mathrm {cv}}$ different, randomly generated 10-fold stratifications. For each stratification and each model, we output the previously described performance evaluation measures (namely, AUC, sensitivity, specificity, F1 score, accuracy) and, for each variable, a measure of its relevance in the risk prediction task.

More precisely, given a risk prediction model RM (that is, RF, AT, or GLM) and an imputation method imp (*miceRF* or *missForest*), producing $m$ imputed sets $D_{\mathrm {imp}}(i)$, $i= 1, \ldots, m$, Rubin’s rule [53], [55] provides a way to combine the “results” (that is either risk prediction performance or the importance of a single variable) computed by the $n_{\mathrm {cv}}$ different runs of each risk prediction model on each of the $m$ imputed sets. Precisely, let $\mathrm {RM}(D_{\mathrm {imp}}(i), \mathrm {fold}(t))$ denote the result computed by RM (e.g., RF importance for a single variable), when using the $t~^{\mathrm{ th}}$ fold stratification, $\mathrm {fold}(t)\,\,(t = 1, \ldots, n_{\mathrm {cv}})$, and the $i~^{\mathrm{ th}}$ imputed set $D_{\mathrm {imp}}(i)\,\,(i = 1, \ldots, m)$. For the sake of simplicity, we organize all $\mathrm {RM}(D_{\mathrm {imp}}(i), \mathrm {fold}(t))$ values in a matrix $\mathrm {RM}(i,t)$ with $m$ rows and $n_{\mathrm {cv}}$ columns. For a fixed imputed set $i$, the mean over the fold stratifications (over the columns of the matrix):

\begin{equation*} \theta (i)=\frac {1}{n_{\mathrm {cv}}}\sum _{t = 1}^{n_{\mathrm {cv}}}{\mathrm {RM}(i, t)}\tag{1}\end{equation*}

is the performance over each $D_{\mathrm {imp}}(i)$, and the mean over all such values is the global result computed using RM and imp:

\begin{equation*} \overline {\theta } = \frac {1}{m} \sum _{i = 1}^{m}{\theta (i)}.\tag{2}\end{equation*}

Rubin’s rule [53], [55] defines the variance of such result by applying the law of total variance [134] to consider both the uncertainty that comes from the processing method applied to each of the imputed datasets (within-imputation variance) and the added uncertainty that comes from the multiply imputed data (between-imputation variance). Precisely, variances computed along each row (over the $n_{\mathrm {cv}}$ values) are the within-imputation variances over each imputed set:

\begin{equation*} \mathrm {Var}(\theta (i)) = \frac {1}{n_{\mathrm {cv}}-1} \sum _{t = 1}^{n_{\mathrm {cv}}}\left ({\mathrm {RM}(i,t) -\theta (i)}\right)^{2}\tag{3}\end{equation*}

while the mean of all the $m$ within-imputation variances is the global within-imputation variance:

\begin{equation*} W = \frac {1}{m} \sum _{i=1}^{m} \mathrm {Var}(\theta (i))\tag{4}\end{equation*}

and the between-imputation variance is the variance of the performance measures achieved over all the imputations:

\begin{equation*} B = \frac {1}{m-1} \sum _{i = 1}^{m}\left ({\theta (i)-\overline {\theta }}\right)^{2}.\tag{5}\end{equation*}

Then the global (normalized) variance associated obtained by imp and RM is computed as [122]:

\begin{equation*} \mathrm {Var}\left ({\theta }\right) = W+\left ({1+\frac {1}{m}}\right)B.\tag{6}\end{equation*}

At the state of the art, MI is used before linear or logistic regression, to determine the coefficients that link predictor variables to a response variable. As reported in [122], for two-sided hypothesis testing of single regression coefficients after MI, the Wald statistic:

\begin{equation*} \mathrm {Wald} = \frac {\overline {\theta }- \theta _{0}}{\mathrm {Var}\left ({\theta }\right)}\tag{7}\end{equation*}

can be used to assess the significance of the difference between the computed estimate $\overline {\theta }$, and the value under the null hypothesis, $\theta _{0}$ (which is generally set to zero), exploiting the fact that Wald follows a chi-square distribution with 1 degree of freedom.

#### 3) Statistical Analysis of Computed Results

Besides computing a global performance measure by applying Rubin’s rule, statistical analysis was applied to compare the performance values computed by different combinations of imputation algorithm and risk prediction approach (Section V). Precisely, we averaged the $n_{\mathrm {cv}}=5$ performance values obtained on each imputed set (we recall that $n_{\mathrm {cv}}$ are the different 10-fold stratifications), thus obtaining $m$ mean values for each imputation $\text {method} + \text {risk}$ prediction approach. At this stage, the one-sided Wilcoxon signed-rank test at the 99% confidence level (p-$\text {value} < 0.01$) was applied to perform the statistical comparison between the distributions of the $m$ mean performance values computed by a combination of imputation algorithm and risk prediction model.

### B. Comparative Evaluation

We started our comparative evaluation by applying the one-sided signed-rank Wilcoxon to compare the performance measures computed when using *miceRF* or *missForest* as the first step for data imputation (see Table 4 in Appendix B). We firstly compared the risk prediction performance measures achieved by the two imputation methods, irregardless of which risk prediction model is used (column “All risk models” in Table 4, Appendix B). Then, we iterated over all risk prediction models, in turn fixing one of them and comparing the performance distribution when using either *miceRF* or *missForest* followed by the fixed risk prediction model (columns “RF”, “AT”, and “GLM” in Table 4, Appendix B). Only the specificities obtained with fixed ATs do not show any statistically significant difference; otherwise, *missForest* always achieved the best result. Therefore, we conclude that in this risk prediction task *missForest* is the most suitable imputation method.

In Table 2 we show the performance measures (and variance) computed by using Rubin’s rule to combine the results computed on the $50 \times 5\,\,10$-fold cross-validation runs performed by each of the three risk-prediction models when using either the datasets imputed by using *missForest* or *miceRF* and the increasing univariate imputation order. For each column in Table 2, the highest global mean, confirmed by the one-sided Wilcoxon signed-rank test (p-values reported in Table 5, Appendix B), is highlighted with bold typeface. The results show that, for what regards the AUC, the sensitivity, the F1-score, and the accuracy, RF is the best performing method, especially when combined with *missForest*. Note that, though no statistically significant difference has been found by one-sided Wilcoxon signed-rank test when comparing the specificity values computed by the three models (see Table 5 in Appendix B), the seemingly lower specificity achieved by RFs both in the comparison with ATs and GLMs is balanced by a higher sensitivity. In practice, both ATs and GLMs are affected by class imbalance, while RFs can cope with such problems by balancing the sampled points during the training phase. Since in our risk prediction model type II errors are worse than type I errors, we can state that the combination of *missForest* + (balanced) RFs is the best performing risk prediction model.

**TABLE 2:** Global Performance Measures Computed by Each Imputation Algorithm + Risk Prediction Model

	model	AUC (var)	Sensitivity (var)	Specificity	F1-score	Accuracy
missForest	RF	0.81 (0.00007)	0.72 (0.00016)	0.76 (0.00006)	0.62 (0.00009)	0.74 (0.00006)
miceRF	AT	0.67 (0.00013)	0.51 (0.00039)	0.83 (0.00020)	0.53 (0.00028)	0.67 (0.00013)
	GLM	0.80 (0.00001)	0.56 (0.00002)	0.86 (0.00001)	0.62 (0.00002)	0.71 (0.00001)
	RF	0.79 (0.00011)	0.70 (0.00034)	0.74 (0.00012)	0.60 (0.0002)	0.72 (0.00014)
	AT	0.65 (0.00027)	0.48 (0.00079)	0.82 (0.00022)	0.50 (0.00062)	0.65 (0.00027)
	GLM	0.78 (0.0005)	0.53 (0.00025)	0.85 (0.00004)	0.59 (0.00014)	0.69 (0.00009)

**TABLE 3:** Ranges of Between-Imputation Variances Achieved by the Four Imputation Methods When Using the Increasing and Decreasing Univariate Imputation Order

Imputation method	Increasing order	Decreasing Order
missForest	2.3E-04 ± 0 [2.3E-04, 2.5E-04]	2.4E-04 ± 0 [1.7E-04, 2.6E-04]
miceRF	1.5E-02 ± 4E-05 [1.3E-02, 1.5E-02]	1.6E-02 ± 1.E-04 [1.5E-02, 2.1E-02]
micePMM	1.4E-02 ± 3E-05 [1.2E-02, 1.4E-02]	1.4E-02 ± 2E-05 [1.3E-02, 1.5E-02]
distFree	3.0E-01 ± 6.1E-03 [4.7E-02, 4.2E-01]	2.5E-01 ± 1.3E-02 [1.0E-02,5.2E-01]

**TABLE 4:** p-Values Resulting From the One-Sided Wilcoxon Signed-Rank Tests Applied to Compare the Performance Values Computed When miceRF or missForest are Used for Imputation

	All risk models		RF		AT		GLM	
Alternative	lower	greater	lower	greater	lower	greater	lower	greater
AUC	1.42E-04	1.00E+00	9.96E-08	1.00E+00	4.40E-03	9.96E-01	4.94E-09	1.00E+00
Sensitivity	2.19E-02	9.78E-01	2.37E-07	1.00E+00	1.18E-02	9.89E-01	7.60E-03	9.93E-01
Specificity	2.67E-02	9.74E-01	1.28E-07	1.00E+00	1.91E-01	8.14E-01	1.43E-04	1.00E+00
F1	7.98E-06	1.00E+00	1.39E-09	1.00E+00	3.53E-03	9.97E-01	6.64E-06	1.00E+00
Accuracy	7.75E-03	9.92E-01	8.06E-08	1.00E+00	4.40E-03	9.96E-01	1.10E-04	1.00E+00

**TABLE 5:** p-Values Obtained by One-Sided Wilcoxon Signed-Rank Test When Comparing the Three Risk Prediction Models

Imputation method	alternative	AUC	Sensitivity	Specificity	F1	Accuracy
missForest	RF vs AT	7.07E-10	6.32E-10	1.00E+00	7.91E-15	7.05E-10
miceRF	RF vs GLM	7.07E-10	6.41E-10	1.00E+00	1.53E-05	7.05E-10
	RF vs AT	7.07E-10	6.32E-10	1.00E+00	7.91E-15	7.05E-10
	RF vs GLM	7.07E-10	6.41E-10	1.00E+00	1.53E-05	7.05E-10

**FIGURE 7.**
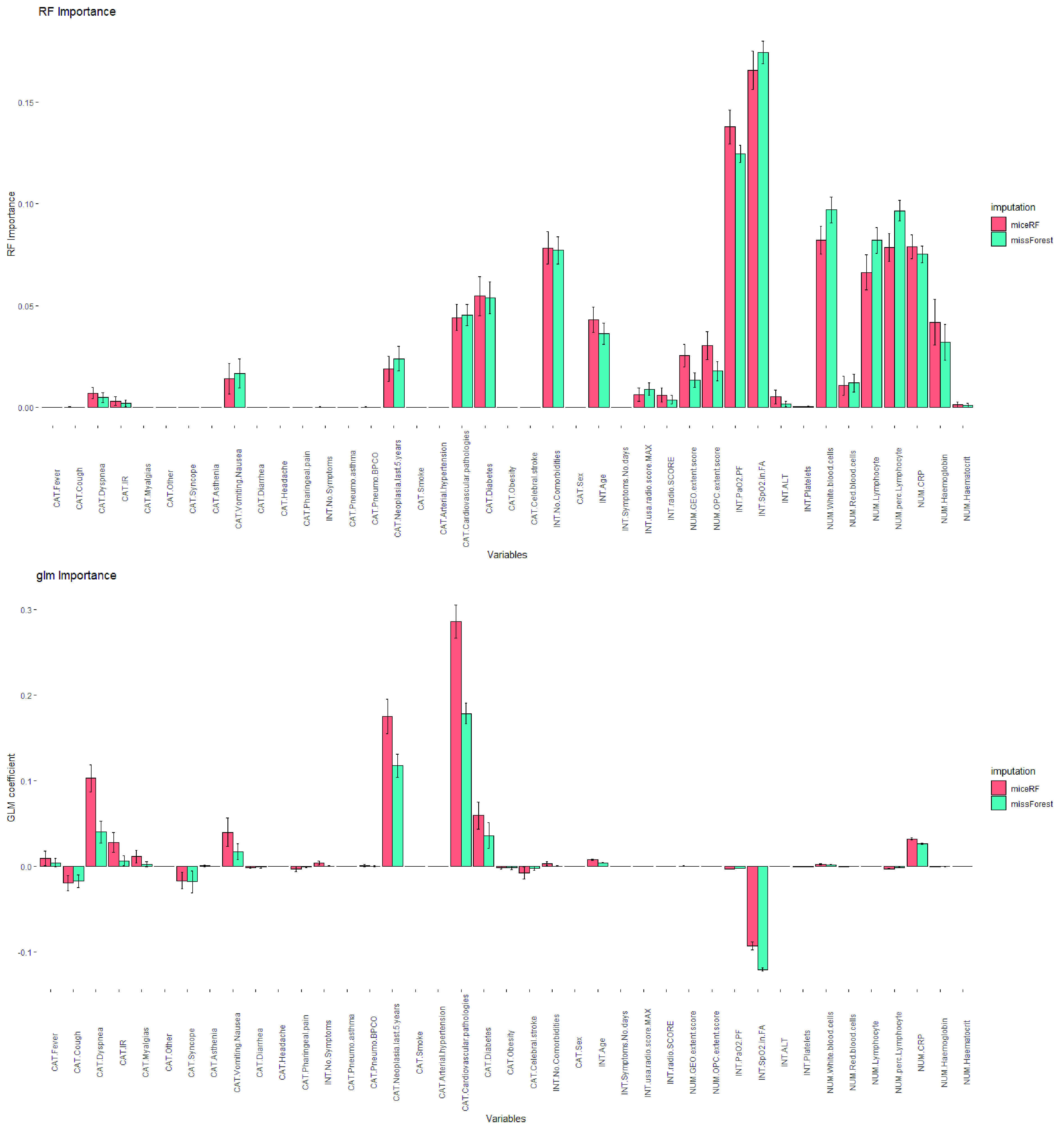
Top: estimates (and standard errors) of the feature relevance computed by RFs. Bottom: estimates (and standard errors) of the feature coefficients computed by GLMs. Only the significant feature relevances/coefficients are plotted.

**FIGURE 8.**
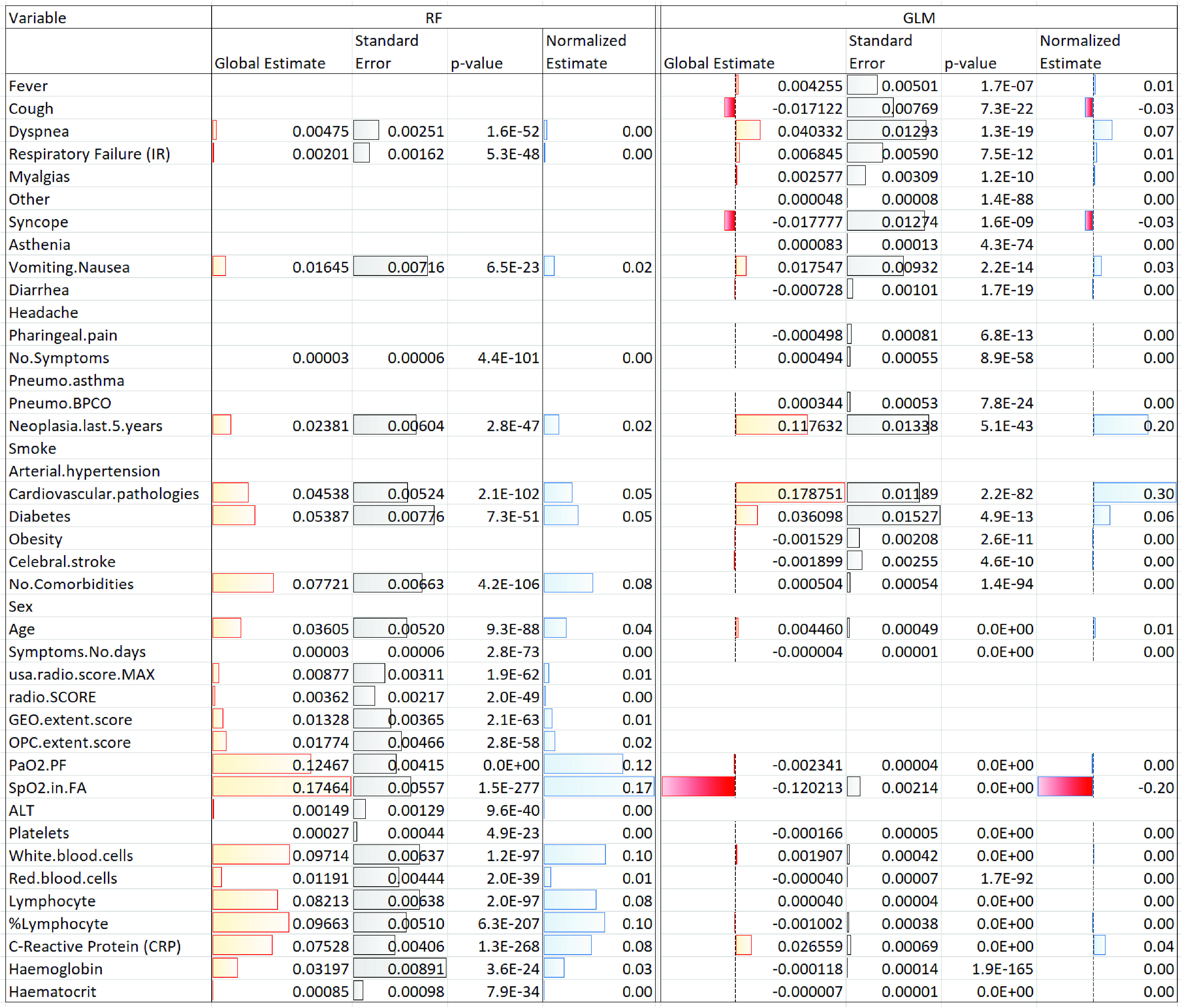
Relevance/coefficient estimates computed by RFs and GLMs. Only the significant estimates are reported. For GLMs, red bars highlight negative coefficients (that is variables, inversely related to the risk).

Finally, since our principal aim is the identification of the most important predictors of severe risk, we analyzed the normalized variable importance computed by RFs, when using either *missForest* or *miceRF* as the preliminary imputation steps. For the sake of comparison, we also considered the coefficients computed by GLMs. After applying Rubin’s rule (see Section VI-A2) to compute, for each feature, the mean (RF) importance or the mean (GLM) coefficient, and their respective variances and standard errors, we applied the Wald significance test to determine the coefficients that were significantly different from zero. The significant RF importance and GLM coefficients, along with their standard errors, are plotted, in the top and bottom panel of Fig. 7, respectively. Fig. 8 reports the precise values of coefficients resulting as significant (Column “Global Estimate”) when using *missForest* followed by RFs (left panel) or GLMs (right panel), together with their standard errors, and the p-values computed by the Wald test. In the visual table in Fig. 8 a column-wise visual comparison of the reported values is allowed by data bars, whose different colors highlight that row-wise comparison is not meaningful. However, to allow a visual comparison of the two global estimates computed by RFs and GLMs, Column “Normalized Estimate” contains the RF variable relevance (left) and GLMs coefficients (right) normalized so that the sum over the column equals one.

Interestingly, the distribution of the feature relevance computed by RFs is very different from that computed by GLMs; generally speaking, RFs mainly consider as relevant all the laboratory variables, the saturation values, and the radiological scores. Even if GLMs predictors selected a similar number of variables (26 variables were selected by GLMs and 25 variables were selected by RFs, see Figs. 7 and 8), and 19 of them are also contained in the subset of variables selected by RFs, the relative importance GLMs attributed to the variables is less balanced. Indeed, GLMs attributed a much higher importance to two comorbidities (cardiovascular pathologies and neoplasia in the last 5 years), followed by only one saturation value (spO2.in.FA), two symptoms (presence of Dyspnea, and Vomiting/Nausea), and only C-Reactive Protein was used among the laboratory variables; the other variables had negligible importance. Such results can be explained by considering that GLMs do not take into account class imbalance; the objective function is easily minimized by decreasing the number of false positives (high specificity), at the expense of a high false negative proportion. Therefore, the features and their relative importance identified by GLMs may be deemed as relevant in the correct identification of patients at low risk. Conversely, the feature selection and importance weighting performed by the proposed RF-based risk prediction system can properly balance sensitivity and specificity.

**FIGURE 9.**
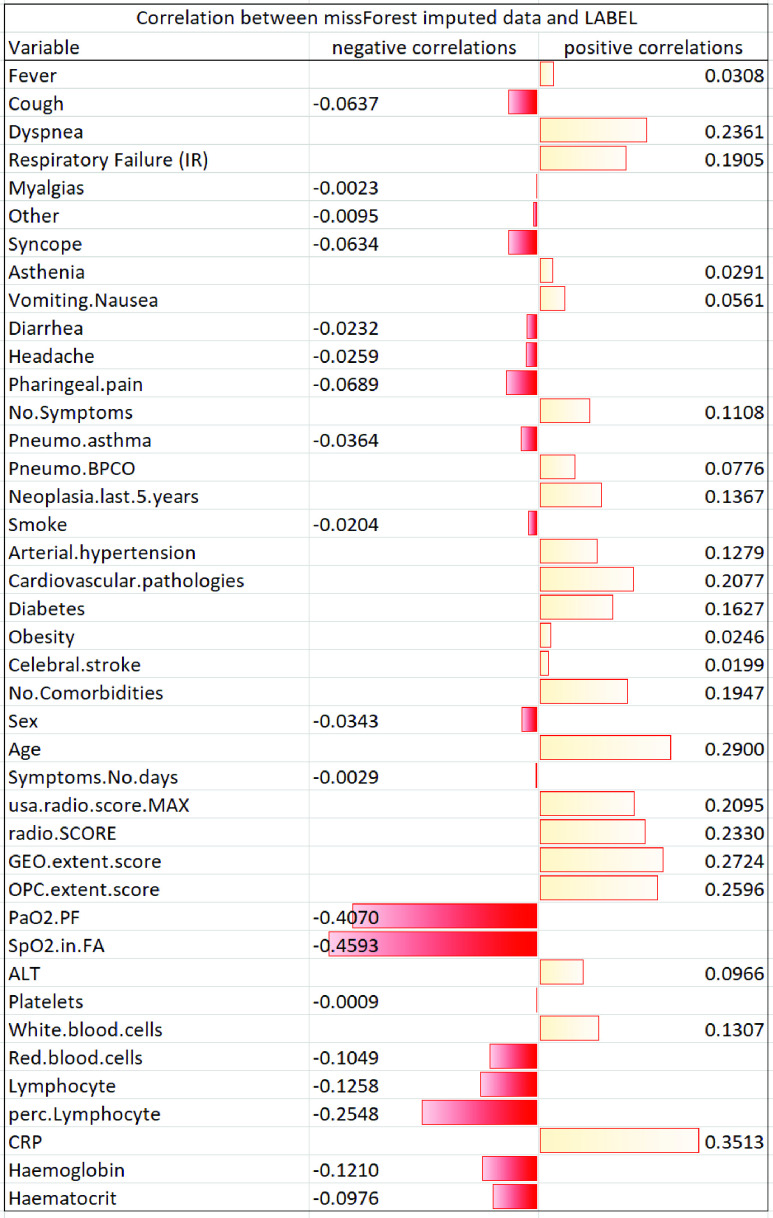
Global, significant estimates of pooled correlation coefficients between each feature and the label computed on the 50 sets imputed by *missForest*.

**FIGURE 10.**
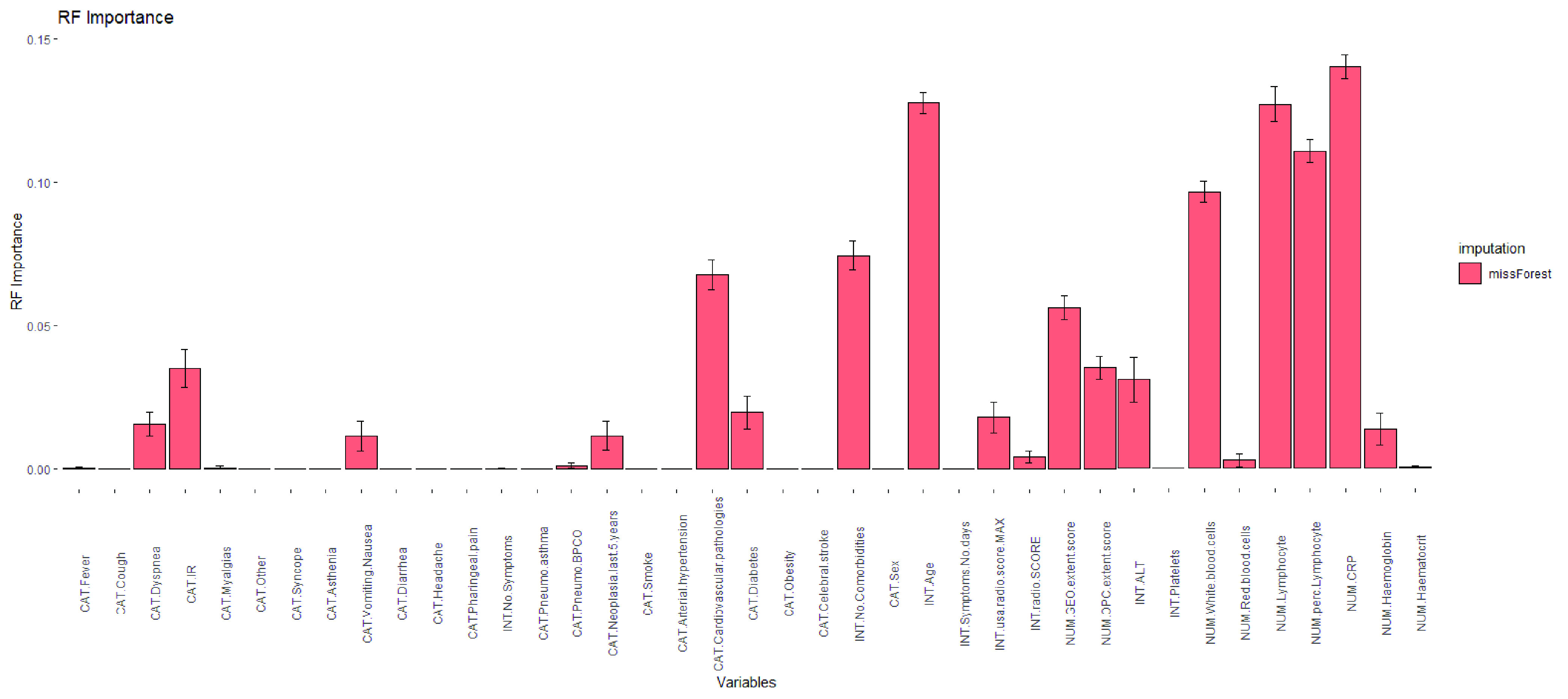
Pooled significant estimates of feature importance computed by RFs on the 50 sets (imputed by *missForest*) when saturation variables are removed.

In sum, we believe that the feature relevance computed through the feature extraction algorithm presented in Section V-A1, followed by the (“balanced”) RFs, is the most reliable. Indeed, the relevant features are similar to those extracted by the papers reported in Table 3 in [15], though none of those works sorted features according to their relevance. We identified the following variables as most relevant (in decreasing order): saturation values (spO2 in free air and paO2.PF), white blood cell counts, lymphocyte counts, the number of comorbidities, C-reactive protein, diabetes, cardiovascular pathologies, age, haemoglobin, neoplasia in the past 5 years, the opacity score computed on CXR by the deep network, nausea, the extent of COVID-19 pattern computed on CXR by the deep network (*OPC.extent.score* and *GEO.extent.score*), the red blood cell count, *usa.radio.score*, dyspnea, *radio.score*, respiratory failure (IR), and haematocrit.

**FIGURE 11.**
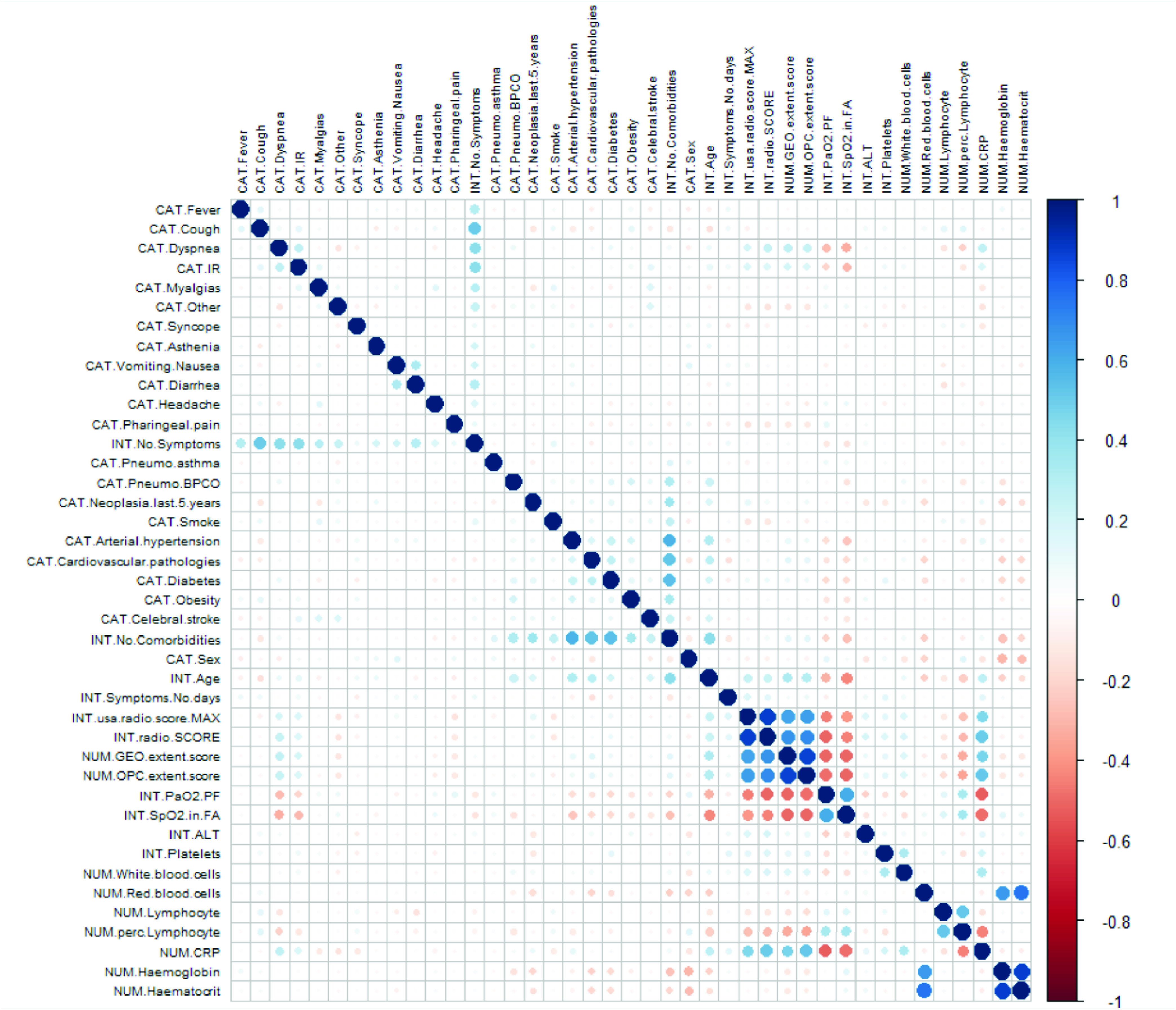
Pooled pairwise (Perason, spearman, and Kendall’s) correlation coefficients between pair of variables computed over the 50 datasets imputed by *missForest*.

Interestingly, the relevance attributed by RFs to radiological features are quite low; moreover, the radiological score computed by deep networks is higher than that computed by experts. To understand such results, we considered the 50 sets imputed with *missForest* and, for each set, we computed the pairwise Pearson, Spearman, and Kendall correlations between features. Subsequently, we used Rubin’s rule in order to pool the mean correlation estimates and to verify their significance (see Fig. 11 in Appendix C). The same procedure was used to compute an estimate of the correlation between each feature and the label (see Fig. 9).

By observing the computed pairwise correlations (in Fig. 11, Appendix C), we note that radiological features are positively correlated (as expected); moreover, they also correlate with C-Reactive Protein (CRP), and have an inverse correlation with the saturation values. Concerning the correlations with the label (Fig. 9), we note that saturation values have the highest (absolute) correlation with the label, followed by CRP, the radiological scores computed by CovidNet, and the radiological scores computed by experts. The obtained correlation results explain the computed relevance; indeed, among a set of correlated features, RFs tend to choose the variables with the highest discriminative power, neglecting the other ones.

As expected, oxygen saturation values are inversely correlated with some symptoms (dyspnea and respiratory failure - IR) and comorbidities (cardiovascular pathologies or arterial hypertension) (Fig. 11, Appendix C). Therefore we performed a test by running all the algorithms without using the two saturation variables (SpO2 in free air - SpO2.in.FA - and PaO2.PF); we retrained RFs (on the features selected as described in Section V-A1) by using 50 MI sets imputed by *missForest*.

With this setting the pooled risk prediction estimates displayed a reduction in accuracy by a mean of 0.06, over the five performance measures (AUC from 0.81 to 0.76, sensitivity from 0.72 to 0.66, specificity from 0.76 to 0.71, F1 score from 0.62 to 0.55, accuracy from 0.74 to 0.68).

The pooled, significant feature-importance estimates are shown in Fig. 10. In this case, CRP is attributed a much higher relative relevance, together with patient’s age. The importance of lymphocyte values, and of all the laboratory variables, is confirmed and radiological features (particularly those computed by CovidNet) have an increased relevance. As expected, those symptoms and comorbidities that are related to saturation values have a significant importance.

## VII. Limitations of the Study

Though promising results were obtained with the proposed risk-prediction system, our study has some limitations.

At first, though we use RF classifiers for the high explainability of their decisions, the complexity of RFs explanations grows with the number of trained trees. For this reason, we propose using ATs, which are derived by the trained RFs to produce a unique, simple, explainable predictor summarising the RF rules. Unfortunately, ATs are not robust with respect to class imbalance. This is because the greedy procedure used to generate ATs iteratively adds the best rule from the RFs, where rule evaluation is measured on all the training set, without normalization with respect to the between-class proportions.

Therefore, even if ATs can provide simple and human understandable decision rules, a limitation of this approach is that the resulting model does not exactly fit the original RFs, and the accuracy is significantly worsened. To deal with this issue, our future work will be therefore aimed at modifying the procedure proposed in [28], [29] in order to obtain ATs robust w.r.t. class imbalance.

With this setting, the features that were considered as most relevant during training were: saturation values, laboratory values (lymphocyte counts, C-Reactive Protein, white blood cells counts, haemoglobin), variables related to comorbidities (number of comorbidities, presence of cardiovascular pathologies and/or arterial hypertension), radiological values computed through CovidNet, and presence of symptoms (vomiting/nausea or dyspnea or respiratory failure).

Another limitation of our study is that the dataset contains only 300 patients and is not public due to privacy restrictions. Since no public dataset with a larger sample size is available yet, the importance of the selected feature set was confirmed by clinical experts, but it has yet to be validated on a larger and more diverse population.

Finally, the limit of the review in [15] and of our work, which stems from the lack of a shared dataset, is that an objective comparative evaluation with state-of-the-art models is not possible. The opportunity for the scientific community to use common datasets is one of the main and important goals to simplify and speed up research activity. In summary, it is necessary to create a deidentified, shareable database to enable an objective comparative evaluation of more rigorous and exhaustively tested prediction models.

## VIII. Conclusion

In this article we pursued the development of a prediction model able to process clinical, radiological, and laboratory data of COVID19-related patients in order to predict their risk of severe outcomes.

The clinical and laboratory values were collected at the time of each patient’s presentation to the ED, while the four radiological values were retrospectively evaluated from the patients CXR, by either pooling radiological experts’ evaluations or by applying CovidNet [11], [14].

The collected variables contain missing values. Therefore, as advocated in [15], we firstly conducted a thorough analysis for identifying both the missingness pattern and the most stable missing data imputation algorithm, among two different MI techniques (*micePMM* and *miceRF*), an RF-based technique (*missForest*), and a maximum-likelihood estimator (*distFree*).

Our evaluation shows that: (i) though the maximum-likelihood imputation method is effective when used for statistically determining whether the data are MCAR or MAR [34], [35], it produces too noisy estimations; (ii) MI techniques reach stability after at least $m = 25$ multiple imputed datasets; (iii) the only method showing negligible between-imputation variance is *missForest*. Our results confirm that, at least $m = 20$ imputed sets should be used for MI to reduce between-imputation variance [35], [125].

Our results demonstrate that stable feature-selection may be obtained by combining the Boruta algorithm and permutation-based feature selection embedded in RFs. When the selected feature set is input to RFs constrained to work on balanced bootstrapped samples, the effect of class imbalance is reduced and improved results are obtained, better than those achieved by either ATs or GLMs. Additionally, we showed that all the risk prediction approaches obtain the best results when using missForest as the previous imputation model.

In conclusion, our analysis demonstrates that the best results are obtained when: (i) imputing the missing data with *missForest*, where the univariate imputation order is based on the increasing amount of missing values, (ii) selecting the most discriminative features by combining Boruta and permutation-based feature selection through an internal cross-validation, and (iii) training RFs on the selected features.

## Appendix A Between-Imputation Variances

In Table 3, the between-imputation variances obtained by the imputation methods *missForest*, *miceRF*, *micePMM*, and *distFree* are reported. As also illustrated in Fig. 5, *missForest* has negligible between-imputation variance, meaning that similar imputations are computed for each missing value. Conversely, *distFree* produces noisier imputations.

## Appendix B Comparative Evaluation Through One-Sided Wilcoxon Signed-Rank Tests

In this appendix we firstly report the p-values for comparisons of the performance evaluation measures computed by using *miceRF* or *missForest* as imputation methods (see Table 4). The column “All risk models” shows the p-values computed when neglecting the separation given by the employed risk prediction models. Columns “RF”, “AT”, and “GLM” report the p-values achieved for RFs, ATs, and GLMs as risk prediction models.

Columns “lower” report the p-value of the one-sided test where the alternative is: “$\textit {miceRF} < \textit {missForest}$”; columns “greater” report the p-value of the one-sided test where the alternative is: “$\textit {missForest} < \textit {miceRF}$”. Note that only the specificities obtained with fixed ATs do not show a statistically significant difference; otherwise *missForest* always achieves the best result.

Next, in Table 5 we report the result of the one-sided Wilcoxon signed-rank test comparing RF vs AT and RF vs GLM, when either *missForest* or *miceRF* are fixed. The p-values express the probability of the null hypothesis when the alternative is “$\text {AT} < \textit {missForest}$” and “$\text {GLM} < \textit {missForest}$”.

## Appendix C Pairwise Correlation Coefficients Between Variables

To visualize the pairwise similarities/dissimilarities between variables distributions, in Fig. 11 we show the pooled correlation coefficients between pairs of variables. These pooled coefficients were computed on the 50 datasets imputed by *missForest* by calculating three pairwise correlation indices (Pearson, Spearman, and Kendall’s coefficients), and by applying Rubin’s rule to pool the $50\times 3$ correlations computed for each pair of variables.

Note that the radiological variables have a relevant and statistically significant (inverse) correlation with saturation values and a high direct correlation with CRP. Such high correlation may be the reason why radiological features obtain a unexpectedly low importance; if two variables have similar distributions, once the RF has used the most discriminating for a split, it will never use the other one for the next splits.

In the plot, each variable name has a prefix that reminds its type; boolean variables have prefix “CAT”, integer variables have prefix “INT”, real variables have prefix “NUM”.
